# TCGA-based identification and functional validation of PBK in intrahepatic cholangiocarcinoma progression with involvement of AKT signaling

**DOI:** 10.1016/j.tranon.2026.102977

**Published:** 2026-08-13

**Authors:** Xiangtian Shi, Lei Zhang, Tengqi Wang, Meng Wang, Chao Wang, Zhangxu Liu, Guixin Zhang

**Affiliations:** a Department of General Surgery, The Second Hospital of Dalian Medical University, Dalian, 116027, Liaoning, China; b Department of General Surgery, Bayannur Hospital, Bayannur, 015000, Inner Mongolia Autonomous Region, China; c Department of Pancreatic Hepato-Biliary-Surgery, The Sixth Affiliated Hospital, Sun Yat-sen University, Guangzhou, 510655, Guangdong, China; d Institute (College) of Integrative Medicine, Dalian Medical University, Dalian, 116044, Liaoning, China

**Keywords:** PBK, AKT signaling, Intrahepatic cholangiocarcinoma, WGCNA, Xenograft

## Abstract

Intrahepatic cholangiocarcinoma (ICC) is an aggressive malignancy with limited molecular targets. This study aimed to identify candidate genes involved in ICC progression and to examine the functional role of PBK. Integrated bioinformatics analysis was performed on TCGA-CHOL data. PBK expression was assessed in ICC clinical samples and cell lines. Gain- and loss-of-function experiments were performed in HUCC-T1 and HCCC-9810 cells. AKT-IN-1 was used to evaluate the involvement of AKT signaling, and preliminary assays were performed to support the use of 1 μM AKT-IN-1 in rescue experiments. A subcutaneous xenograft model was used for in vivo validation. DEG analysis revealed distinct expression profiles between CHOL and non-tumor tissues, and WGCNA identified three tumor-associated modules: METurquoise, MEblue, and MEyellow. PBK was selected for further validation based on its upregulation, survival association, kinase-related background, and limited characterization in ICC. PBK expression was increased in ICC tissues and cell lines and was associated with TNM stage and shorter OS and DFS in the clinical cohort. Internal ROC analysis provided only an exploratory estimate of discriminative ability in the TCGA-CHOL cohort, and this finding requires validation in independent cohorts. PBK knockdown reduced ICC cell proliferation, migration, and invasion, whereas PBK overexpression enhanced these phenotypes. PBK overexpression increased p-AKT (Ser473), and AKT-IN-1 attenuated PBK-induced colony formation, EdU incorporation, migration, and invasion. In vivo, PBK knockdown reduced xenograft growth and was accompanied by decreased p-AKT, KI67, and PCNA expression. These findings suggest that PBK contributes to ICC malignant phenotypes, with AKT signaling involved in this process.

## Highlights

• Integrated TCGA analysis via DESeq2, edgeR, limma & WGCNA identified PBK as key ICC gene.
• PBK overexpressed in ICC, correlates with tumor stage.
• PBK promotes ICC progression via AKT pathway activation; inhibitor abrogates oncogenic effects.
• PBK knockdown suppresses xenograft growth with reduced p-AKT, KI67 and PCNA expression.

## Graphical abstract

**Figure:**
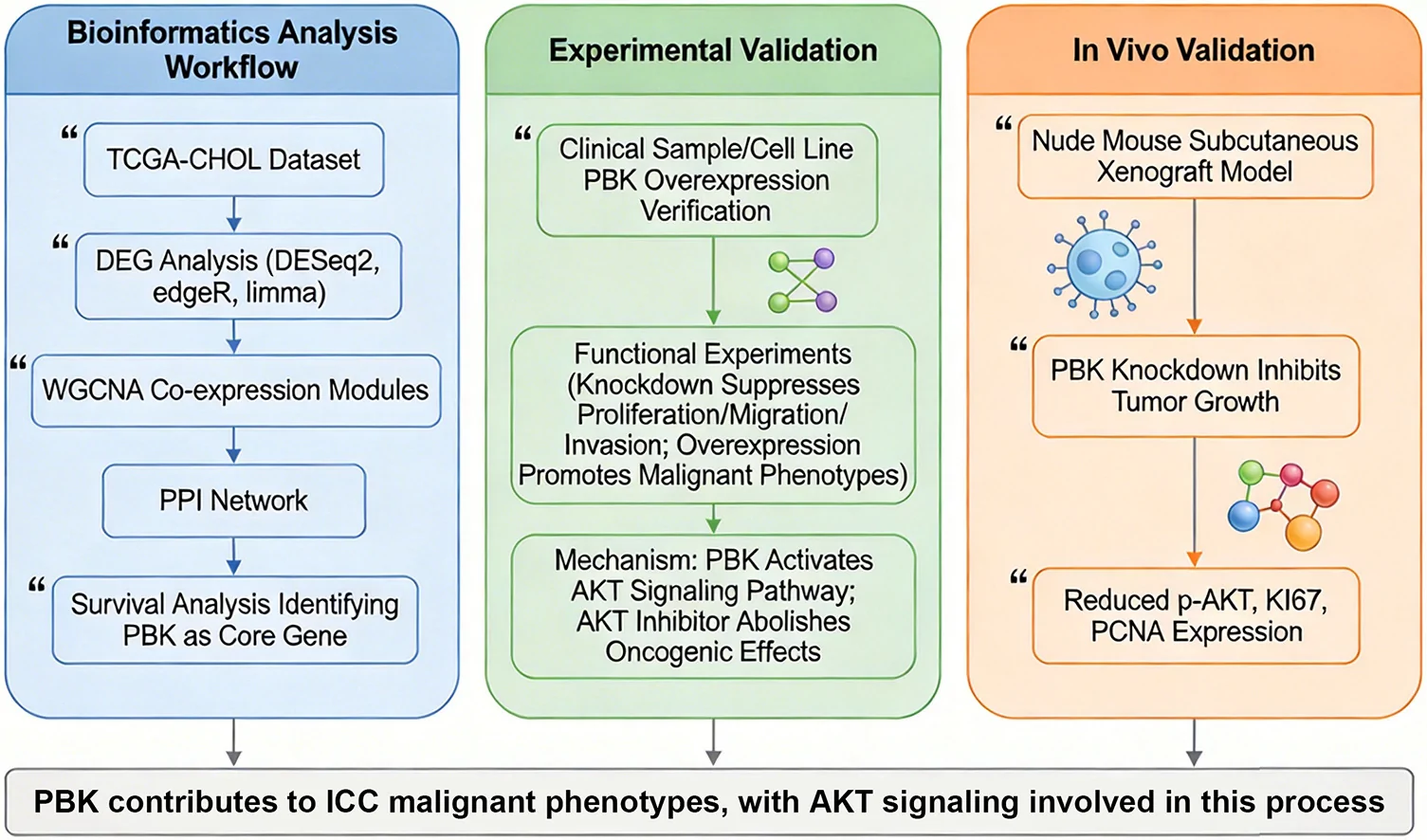

## Introduction

Cholangiocarcinoma (CHOL) is a malignant tumor arising from biliary epithelial cells and is classified into intrahepatic CHOL (ICC), perihilar CHOL, and distal CHOL according to anatomical location [1,2]. ICC originates from the intrahepatic bile ducts and is characterized by aggressive progression and poor prognosis [3]. Although surgical resection remains the main potentially curative treatment for ICC, many patients are diagnosed at an advanced stage and are not suitable for curative surgery [4]. Additionally, the response of advanced ICC to conventional radiotherapy and chemotherapy remains limited, contributing to unsatisfactory long-term survival [5]. Therefore, identifying molecular alterations associated with ICC progression is important for improving disease understanding and for exploring molecules with potential biological and clinical relevance.

With the development of high-throughput sequencing and bioinformatics, public transcriptomic datasets have provided useful resources for investigating tumor-related molecular changes [6]. The TCGA database contains genomic, transcriptomic, and clinical information and has been widely used for differential expression analysis, pathway exploration, and prognostic evaluation [7,8]. However, single analytical approaches, such as DEG analysis alone, may generate a large number of candidate genes, including false-positive or background signals [9]. Integrated strategies combining DEG analysis, weighted gene co-expression network analysis (WGCNA), and protein-protein interaction (PPI) network analysis may help prioritize candidate genes that are more closely associated with tumor status and clinical outcomes [10,11]. Nevertheless, candidate genes identified from public datasets require experimental validation in disease-relevant models to confirm their biological relevance.

PBK, also known as TOPK, is a serine/threonine kinase that has been implicated in tumor cell proliferation, migration, invasion, and cell-cycle regulation in several malignancies [[12], [13], [14], [15]]. Although PBK/TOPK has been investigated in different cancer types, its functional role in ICC progression and its relationship with AKT signaling remain insufficiently defined. These observations support further investigation into the functional role of PBK in ICC and its possible association with AKT signaling.

Based on these considerations, the present study analyzed the TCGA-CHOL dataset using multiple bioinformatics approaches, including DEG analysis, WGCNA, and PPI network analysis, to screen candidate genes associated with CHOL progression. PBK was selected for further validation based on its upregulation, association with survival, kinase-related biological background, and limited functional characterization in ICC. We then examined PBK expression in ICC clinical samples and cell lines and evaluated its effects on ICC cell proliferation, migration, and invasion using gain- and loss-of-function experiments. In addition, AKT-IN-1 rescue experiments and a xenograft model were used to explore the involvement of AKT signaling in PBK-regulated ICC phenotypes.

## Materials and methods

### Data source and preprocessing

The CHOL cohort was derived from the GDC TCGA Bile Duct Cancer (TCGA-CHOL) project through the UCSC Xena Browser (https://xenabrowser.net/datapages/). The TCGA-CHOL cohort was used for public database screening. Clinical sample validation, cell-line experiments, and animal experiments were focused on intrahepatic cholangiocarcinoma (ICC). In this study, “TCGA-CHOL” or “CHOL” refers to database-based analyses, whereas “ICC” refers to the validation cohort and experimental models. The downloaded data included: (1) RNA-seq expression data; (2) clinical phenotype data; and (3) survival data. Count-based expression data were used for DESeq2, edgeR, and limma-voom analyses. Log2-transformed expression matrices from UCSC Xena were used for visualization and expression-based downstream analyses when applicable. A total of 45 samples were included in the database analysis, including 36 tumor samples and 9 non-tumor samples. Sample grouping was based on the 14th–15th digits of the TCGA barcode: values < 10 were classified as tumor samples, and values ≥ 10 as non-tumor samples.

### DEG analysis

Three widely used bioinformatics tools — DESeq2, edgeR, and limma-voom — were employed for differential expression analysis. All three methods utilized the same filtered count matrix as input, with the grouping variable specified as “condition” (tumor vs normal). Based on the negative binomial distribution, DESeq2 applies a generalized linear model to determine size factors for normalization, and Wald tests were used to calculate differential expression statistics. edgeR involves constructing a DGEList object followed by trimmed mean of M-values (TMM) normalization; after estimating dispersions and fitting generalized linear models (GLMs), likelihood ratio tests (LRT) were applied to identify DEGs. For limma-voom, count data were subjected to voom transformation to model the mean-variance relationship, and differential expression was determined using linear models combined with empirical Bayes methods. Criteria for filtering differential genes was established as |log_2_ fold change (log_2_FC)| > 1 and *P*〈 0.05. Among genes meeting the threshold with log_2_FC 〉 1 were defined as upregulated (UP), those with log_2_FC < −1 as downregulated (DOWN), and the remainder as non-significant (NOT). Results from each method were exported as DESeq2_DEGs.csv, edgeR_DEGs.csv, and limma_DEGs.csv, respectively. To enhance the robustness of candidate genes and mitigate biases associated with a single method, DEGs consistently identified by all three tools were selected as a high-confidence DEG set: intersections of UP and DOWN genes from the three methods were separately computed to obtain consensus upregulated and downregulated genes, which were used for subsequent functional and network analyses. Principal component analysis (PCA) was utilized with “prcomp” function in R software to assess sample expression heterogeneity. The “VennDiagram” package was applied to construct Venn diagrams for identifying shared upregulated and downregulated genes among the three tools. Heatmaps were constructed to visualize the expression patterns of common DEGs, validating their expression consistency.

### WGCNA

WGCNA R package was utilized to analyze global gene expression data and build a co-expression network. To assess correlations between gene expression patterns among CHOL samples, a pairwise Pearson correlation matrix was established. Subsequently, the similarity matrix was converted into an adjacency matrix using the following formulas: Sij = |cor(xi, xj)| and aij = Sij^β, where Sij represents the expression similarity between genes xi and xj, aij denotes the element of the adjacency matrix, and β is the soft-thresholding power. Based on the scale-free network property, the soft-thresholding power β was determined using the soft connectivity function in the WGCNA package. The scale-free topology fit index and mean connectivity were evaluated under different soft-thresholding powers, and β = 5 was selected for subsequent network construction. The corresponding diagnostic plots are presented in Supplementary Fig. S1. The topological structure of the gene co-expression network was considered to approximate a scale-free network when the topological fit index reached 0.85 or higher at a relatively low power. Network modules were characterized using the topological overlap matrix and its corresponding dissimilarity matrix, where the minimum module size was set to 50 and other parameters were kept as default settings. Following the calculation of the first principal component for each module, namely the module eigengene, the association between individual modules and clinical phenotypes was examined. Hub genes within each module were identified according to gene significance and module membership, using cutoff values of GS > 0.5 and MM > 0.8, respectively. Ultimately, genes exhibiting both high MM and high GS values were designated as core genes for subsequent integrated analyses.

### PPI network construction, topology analysis and bioinformatics verification

The consensus DEGs were intersected with the significant genes from phenotype-associated modules in WGCNA to obtain a candidate gene set (common_genes) for constructing the PPI network. These candidate genes were submitted to the STRING database (species restricted to Homo sapiens), and interaction relationships were filtered at medium confidence (combined score ≥ 0.4) with edge lists (TSV format) exported. Subsequently, the PPI network was imported into Cytoscape for visualization and topological analysis. NetworkAnalyzer was used to calculate network parameters (e.g., Degree, Betweenness centrality, Closeness centrality, etc.), and the results were saved as PPI_result.csv. Genes were ranked using node Degree as the primary criterion, and the top 50 genes with the highest Degree were defined as hub genes for further downstream analyses, including Kaplan-Meier survival analysis and internal ROC analysis. Kaplan-Meier survival analysis was performed using the “survival” and “survminer” packages in R software, and the log-rank test was used to assess the association between gene expression and OS in the TCGA-CHOL cohort. Genes with *P* < 0.05 were considered survival-associated genes. Receiver operating characteristic curves were plotted using the “pROC” package, and the area under the curve was calculated. AUC values from the TCGA-CHOL cohort were reported as internal ROC estimates.

### Collection of clinical samples

Clinical ICC samples were collected from patients who underwent surgical resection, and all cases were pathologically confirmed as ICC. The clinical samples were used for two separate analyses. First, three paired ICC tissues and adjacent non-tumor tissues were used only for Western blot validation of PBK protein expression and were not included in the clinicopathological correlation or survival analyses. Second, tumor tissues from an independent cohort of 38 ICC patients were used for RT-qPCR-based PBK mRNA expression analysis, clinicopathological correlation analysis, and survival analysis. For this 38-patient cohort, PBK mRNA expression was quantified by RT-qPCR, normalized to GAPDH, and calculated using the 2^-ΔΔCt method. The tumor-to-adjacent non-tumor expression ratio of PBK was then used for clinical stratification. Patients with a PBK expression ratio ≥2.51 were classified as the PBK-high group, whereas those with a ratio <2.51 were classified as the PBK-low group, resulting in 22 patients in the PBK-high group and 16 patients in the PBK-low group. Exclusion criteria included non-ICC diagnosis by pathological examination and insufficient clinical documentation. Informed written consent was obtained from all participating patients, and this study was approved by the Bayannur Hospital Committee (approval number 20251107002). All procedures involving human participants were performed in accordance with the ethical standards of the institutional and/or national research committee and the 1964 Helsinki Declaration and its later amendments.

### Correlation of clinical pathological parameters and survival analysis

In the 38-patient RT-qPCR cohort, the correlation between PBK expression status and clinicopathological characteristics was examined using the Chi-square test or Fisher’s exact test, as appropriate. Kaplan-Meier survival curves were constructed using the same cohort to evaluate the relationships between PBK expression status and overall survival (OS) as well as disease-free survival (DFS), and differences were assessed using the log-rank test.

### Cell culture and treatment

Human intrahepatic biliary epithelial cells (HIBEC, CTCC-034-HUM) were purchased from MEISEN CELL. Human ICC-derived cell lines HUCC-T1 (TCHu281), HCCC-9810 (TCHu17), and RBE (SCSP-557) were obtained from the Shanghai Institute for Biological Science. Cells were cultured in RPMI 1640 medium supplemented with 10% FBS at 37 °C in a humidified incubator containing 5% CO₂. Stable PBK-knockdown and PBK-overexpressing cell lines were generated in both HUCC-T1 and HCCC-9810 cells by Guangzhou IGE Biotechnology Co., Ltd. The knockdown groups included HUCC-T1-shNC, HUCC-T1-shPBK, HCCC-9810-shNC, and HCCC-9810-shPBK. The overexpression groups included HUCC-T1-LVNC, HUCC-T1-LVPBK, HCCC-9810-LVNC, and HCCC-9810-LVPBK. For AKT-IN-1 rescue experiments, HCCC-9810-LVPBK cells were treated with 1 μM AKT-IN-1 (HY-18296, MCE). The experimental groups were HCCC-9810-LVNC, HCCC-9810-LVPBK, and HCCC-9810-LVPBK + AKT-IN-1. AKT-IN-1 was maintained during the corresponding functional assays. To assess the appropriate working concentration of AKT-IN-1, HCCC-9810 cells were treated with 0, 0.1, 0.3, 1, or 3 μM AKT-IN-1 for 48 h, followed by CCK-8 analysis. HCCC-9810 cells treated with 1 μM AKT-IN-1 for 48 h were further used for Western blot analysis of p-AKT (Ser473), p-ERK1/2, and p-p38 (Thr180).

### Real-time quantitative polymerase chain reaction (RT-qPCR)

RT-qPCR assay was utilized to determine PBK levels. Total RNA was first isolated using the TriQuick Reagent total RNA extraction kit (R1100, Solarbio), followed by reverse transcription of RNA into complementary DNAs (cDNAs) using 5 × RT SuperMix for qPCR (K1074, APEXbio). The relative expression level of PBK was quantified using 2 × SYBR Green qPCR Master Mix (K1070, APEXbio) on an iQ5 Multicolor Real-Time PCR Detection System (582BR 005500, BIO-RAD). PBK expression was calculated using the 2^−ΔΔCt^ method, with GAPDH serving as the internal control. The primers are listed as follows: PBK-F: 5′-TATGACTGCTCCTGCCTTCATAAC-3′, PBK-R: 5′-CACAGCTTCTTTGGGTTTCCAT-3′; GAPDH-F: 5′-TCTCTGCTCCTCCCTGTTC-3′, GAPDH-R: 5′-ACACCGACCTTCACCATCT-3′.

### Western blot

Western blot was conducted to assess PBK, p38, p-p38 (Thr180), ERK1/2, p-ERK1/2, AKT, p-AKT (Ser473), KI67, and PCNA levels. Total proteins were isolated with RIPA buffer (P0013B, Beyotime). After quantification using a BCA protein assay kit (BL521A, Biosharp), 20 μg of total protein per sample was loaded onto 12% polyacrylamide gels for separation. Proteins were separated by SDS-PAGE and transferred to PVDF membranes. Membranes were blocked in 5% nonfat milk, followed by incubation at 4 °C overnight with primary antibodies against PBK (ab236872, Abcam), p38 (ab170099, Abcam), p-p38 (Thr180, ab178867, Abcam), ERK1/2 (ab184699, Abcam), p-ERK1/2 (ab201015, Abcam), AKT (ab32505, Abcam), p-AKT (Ser473, ab38449, Abcam), KI67 (ab92742, Abcam), PCNA (ab92552, Abcam), and GAPDH (ab181602, Abcam). Thereafter, membranes were incubated with HRP-conjugated secondary antibodies (BL003A/BL001A, Biosharp). Finally, membranes were treated with ECL chemiluminescence reagent (K-12045-D50, Advansta), and target protein bands were visualized using a ChemiScope6100 imaging system (CLiNX). Band intensities were quantified using ImageJ software. Phosphorylated proteins were normalized to their corresponding total proteins, including p-AKT/AKT, p-ERK1/2/ERK1/2, and p-p38/p38. PBK, KI67, and PCNA were normalized to GAPDH.

### Cell counting kit 8 (CCK-8) assay

Cell viability was evaluated using a CCK-8 kit (CA1210, Solarbio). HUCC-T1 and HCCC-9810 cells from the indicated groups were rinsed with PBS, trypsinized with 0.25% trypsin (25200–072, Gibco), and resuspended in complete medium. After counting, cells were adjusted to 3 × 10⁴ cells/mL and seeded into 96-well plates at 100 μL per well. Three replicate wells were prepared for each group at each time point. At 12, 24, 48, 72, and 96 h after seeding, 10 μL of CCK-8 solution was added to each well, followed by incubation for another 2 h at 37 °C. Absorbance was measured at 450 nm using a microplate reader. Cell viability after AKT-IN-1 treatment was assessed using the same CCK-8 procedure.

### Clone formation assay

HUCC-T1 and HCCC-9810 cells from the indicated groups were seeded in 6-well plates or culture dishes at 400 cells per well. Following the respective treatments, cells were cultured for 7 days until macroscopically visible colonies formed. Subsequently, cells were rinsed with PBS and incubated with 0.5% crystal violet staining solution (dissolved in 2% ethanol) for 15–30 min. Unbound dye was removed by thorough rinsing with deionized water. Finally, colonies with a diameter greater than 2 mm were enumerated using a light microscope, and colony formation efficiency (CFE) was computed via the following formula: CFE = (total number of colonies / initial number of seeded cells) × 100%.

### EDU assay

EdU DNA Proliferation in vitro Detection Kit (C10310, RiboBio) was utilized to assess the proliferation of HUCC-T1 and HCCC-9810 cells at 48 h. Cells were cultured in medium with 50 μM EdU for 24 h, followed by rinsing with PBS. Cells were fixed with 4% paraformaldehyde supplemented with 2 mg/mL glycine. Subsequently, cells were treated with 1 × Apollo® staining solution under dark conditions for 30 min, prior to final staining with 1 × Hoechst 33342 solution. After staining, EdU-positive cells were counted using a fluorescence microscope.

### Transwell assays

For cell migration assays, HUCC-T1 and HCCC-9810 cells were rinsed with PBS, followed by trypsinization with 0.25% trypsin to obtain single-cell suspensions. Cells were centrifuged at 800 rpm for 5 min. After aspirating the supernatant, cells were reconstituted in basic medium, counted, and standardized to a final concentration of 1 × 10⁶ cells/mL. A total of 100 μL of cell suspension was added to the upper chamber of Transwell inserts, while 600 μL of complete medium was added to the lower chamber. After incubation for 72 h, the inserts were collected. Residual cells remaining in the upper chamber were gently removed using sterile cotton swabs. Migrated cells were fixed with 4% paraformaldehyde for 15 min and stained with 1% crystal violet for 10 min. Images were captured under a microscope, and the number of migrated cells was counted.

For cell invasion assays, Matrigel (BD 356234, BD Biocoat) was utilized, with all other steps identical to those described above for migration assays. Briefly, Matrigel was rehydrated overnight at 4 °C and diluted in pre-cooled serum-free medium at a 1:3 dilution ratio. Forty microliters of the diluted Matrigel was dispensed into pre-chilled Transwell chambers and incubated at 37 °C for 2 h to facilitate gel formation. Cells were then seeded and incubated for 72 h before fixation, staining, imaging, and counting.

### Subcutaneous xenograft model in nude mice

Six-week-old male BALB/c nude mice were housed in an SPF animal facility, maintained at 22±2 °C with 50±5% humidity and a 12 h light/12 h dark cycle. Following a 1-week acclimation period, the mice were used for experiments. HUCC-T1-shNC and HUCC-T1-shPBK cells (3 × 10⁶ cells/mouse) were suspended in 100 μL of a 1:1 mixture of PBS and Matrigel and subcutaneously injected into the right flank of nude mice. The mice were divided into two groups: HUCC-T1-shNC group and HUCC-T1-shPBK group, with 6 mice per group. Tumor volume was measured on days 7, 14, 21, and 28 after injection using a vernier caliper, and tumor volume was calculated using the formula: V = (long diameter × short diameter²)/2. Four weeks after injection, the mice were humanely euthanized via cervical dislocation, and tumors were rapidly dissected and weighed. Tumor tissues were immediately flash-frozen in liquid nitrogen for subsequent Western blot assays. All animal experiments were approved by the Guangzhou Forevergen Medical Laboratory Animal Center Committee (approval number: IACUC-AEWC-F251015045, dated October 15, 2025). Animal experiments were performed in accordance with ARRIVE guidelines and the IACUC Handbook (third edition).

### Statistical analysis

All experimental data were statistically analyzed using GraphPad Prism 9.0 and R 4.2.1 software and are expressed as the mean ± standard deviation (SD). In vitro experiments were performed using three independent experiments. Student’s *t*-test was used to compare two groups, whereas one-way analysis of variance was applied for comparisons among multiple groups. Associations between PBK expression and clinicopathological parameters were analyzed using the Chi-square test or Fisher’s exact test. Survival curves were generated using the Kaplan-Meier method and compared using the log-rank test. ROC analysis in the TCGA-CHOL cohort was considered an internal exploratory analysis. *P* < 0.05 was deemed statistically significant.

## Results

### DEG analysis in CHOL samples using various tools

**Fig. 1:**
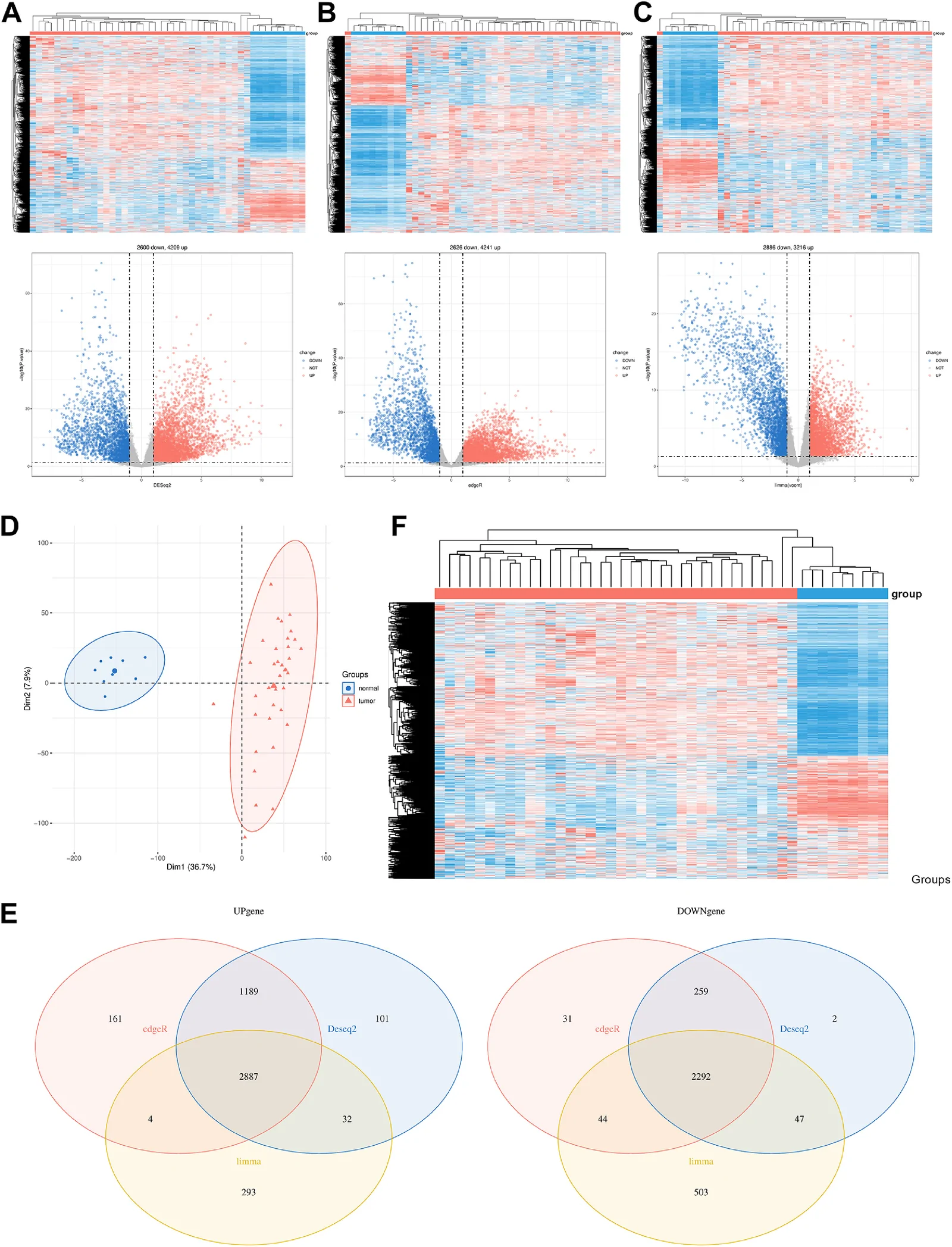
DEG analysis of TCGA-CHOL samples using multiple bioinformatics tools. A. Heatmap and volcano plot derived from DESeq2 analysis. The heatmap presents the expression profile of differentially expressed genes between tumor and non-tumor samples. In the volcano plot, blue dots represent downregulated genes (*n* = 2600), and red dots represent upregulated genes (*n* = 4209). B. Heatmap and volcano plot generated by edgeR, identifying 2626 downregulated genes and 4241 upregulated genes. C. Heatmap and volcano plot generated by limma, identifying 2886 downregulated genes and 3216 upregulated genes. D. PCA displaying the distribution of tumor and non-tumor samples in the TCGA-CHOL cohort. E. Venn diagrams depicting the overlap of upregulated and downregulated genes identified by DESeq2, edgeR, and limma, including 2887 common upregulated genes and 293 common downregulated genes. F. Heatmap summarizing the expression pattern of the common differentially expressed genes.

Initially, we conducted DEG analysis to identify genes with differential expression in CHOL samples relative to controls. For this purpose, we employed three distinct bioinformatics tools: DESeq2, edgeR, and limma. These tools enabled us to identify genes with significant expression differences between CHOL and control groups. Fig. 1A–C illustrated the heatmaps and volcano plots generated from the analyses using DESeq2, edgeR, and limma, respectively. Specifically, DESeq2 analysis identified 2600 downregulated genes and 4209 upregulated genes; edgeR analysis detected 2626 downregulated genes and 4241 upregulated genes; and limma analysis revealed 2886 downregulated genes and 3216 upregulated genes. These findings indicated substantial gene expression differences in CHOL samples, with a greater number of upregulated genes compared with downregulated genes. PCA further illustrated the distribution of CHOL samples at the gene expression level (Fig. 1D). Venn diagram analysis demonstrated that 2887 upregulated genes and 293 downregulated genes were commonly detected by all three tools (DESeq2, edgeR, and limma) (Fig. 1E). The heatmap depicting the expression patterns of these shared genes further presented their distribution across tumor and non-tumor samples (Fig. 1F). Genes consistently identified by all three methods were considered high-confidence DEGs and were used for subsequent integrated analyses.

### WGCNA of CHOL samples in the TCGA database

**Fig. 2:**
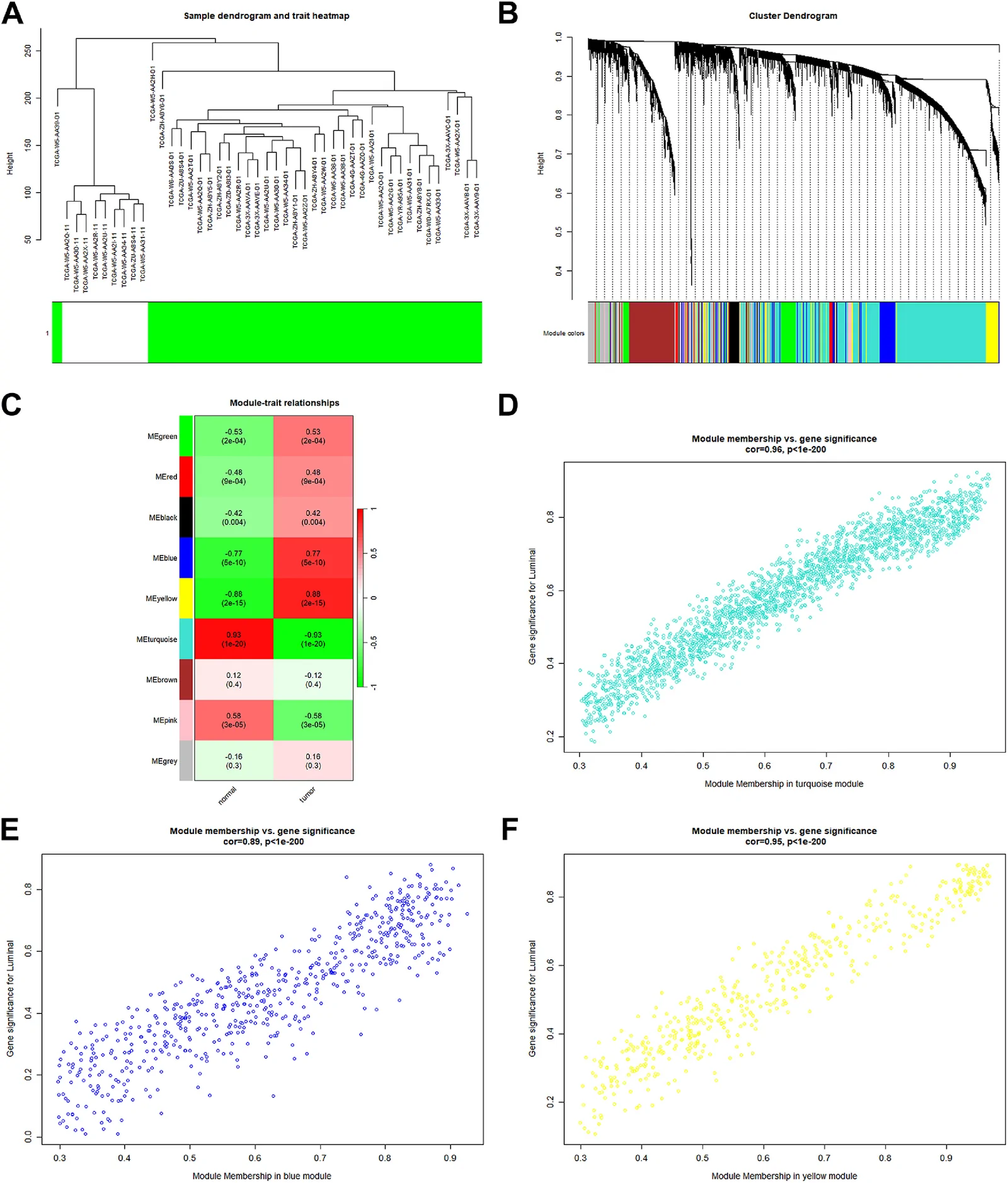
WGCNA of TCGA-CHOL samples. A. Hierarchical clustering illustrating the similarity among samples. The shorter the branch distance, the more analogous the gene expression patterns of the samples. This validates the rationality of the grouping strategy in the experimental design (e.g., whether the normal group and tumor group are distinctly separated). B. Hierarchical clustering based on gene expression correlations was performed to partition genes into co-expression modules (color-labeled). Genes within the same module exhibit coordinated expression patterns, with each color representing a unique module (e.g., MEred, MEblue). Genes in the same module are likely involved in shared biological pathways. C. Heatmap displaying the correlation coefficients (represented by colors) and p values (in parentheses) between each module (rows) and clinical phenotypes (columns, e.g., normal/tumor). D. The relationship between the METurquoise module and tumor status. E. Association of the MEblue module with tumor status. F. Correlation between the MEyellow module and tumor status.

Subsequently, we performed WGCNA on CHOL samples from the TCGA database to identify co-expressed gene modules associated with CHOL and to explore the relationships between these modules and clinical phenotypes. Before module construction, the scale-free topology fit and mean connectivity were evaluated under different soft-thresholding powers, and a soft-thresholding power of β = 5 was selected for subsequent WGCNA (Supplementary Fig. S1A–S1B). Fig. 2A illustrated the results of hierarchical clustering analysis among samples. This analysis grouped the samples into distinct clusters based on their expression patterns, with normal and tumor groups displaying clear separation. Fig. 2B presented hierarchical clustering based on gene expression correlation, which partitioned genes into multiple co-expression modules. Each color corresponds to a module, where genes within the same module exhibit coordinated expression patterns. The heatmap displayed the correlation coefficients and P values between each module and clinical phenotypes (Fig. 2C). Specifically, the METurquoise module showed a strong positive correlation with tumor status (*r* = 0.93, *P* = 1e-20) (Fig. 2D). The MEblue module presented a negative correlation with tumor status (*r* = −0.77, *P* = 5e-10) (Fig. 2E). Similarly, the MEyellow module demonstrated a strong positive association with tumor status (*r* = 0.88, *P* = 2e-15) (Fig. 2F). Based on these module-trait relationships, METurquoise, MEblue, and MEyellow were selected for subsequent integrated analysis.

### Analysis of key genes associated with CHOL and their clinical relevance

**Fig. 3:**
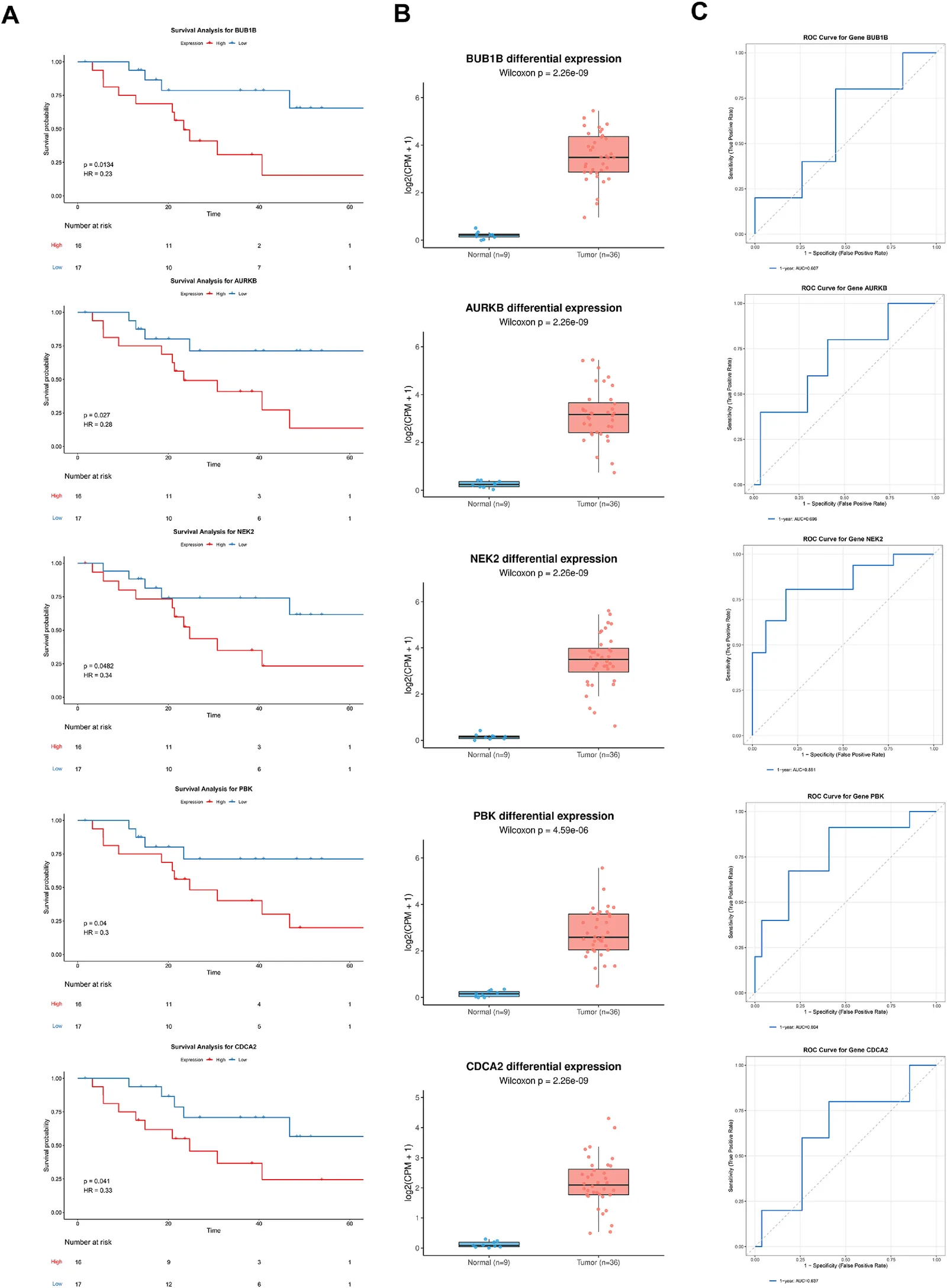
Bioinformatic screening and internal evaluation of candidate genes in the TCGA-CHOL cohort. A. Kaplan-Meier survival analysis of BUB1B, AURKB, NEK2, PBK, and CDCA2 in the TCGA-CHOL cohort. B. Differential expression of BUB1B, AURKB, NEK2, PBK, and CDCA2 between tumor and non-tumor samples in the TCGA-CHOL cohort. Box plots show the median and interquartile range. C. ROC curve analysis of BUB1B, AURKB, NEK2, PBK, and CDCA2 based on the TCGA-CHOL cohort. These ROC curves represent internal exploratory estimates from the discovery dataset.

Furthermore, we intersected the DEGs from the TCGA-CHOL dataset with the hub genes identified through WGCNA to obtain a set of common genes (Table S1). We subsequently performed PPI network analysis on these common genes, and the results were documented in PPI_result.csv (Table S2). After ranking genes according to their degree of connectivity, the top 50 genes were selected for survival analysis. Among these genes, higher expression of BUB1B, AURKB, NEK2, PBK, and CDCA2 was associated with unfavorable survival in the TCGA-CHOL cohort (Fig. 3A). We also examined the expression of these genes between tumor and non-tumor samples. NEK2 and PBK showed prominent upregulation in CHOL tissues relative to non-tumor controls (Fig. 3B). ROC curve analysis was further performed for these genes in the TCGA-CHOL cohort. In this discovery dataset, NEK2 and PBK yielded AUC values greater than 0.8 in the internal ROC analysis (Fig. 3C). Although NEK2 also met several bioinformatic screening criteria, NEK2 has previously been investigated in cholangiocarcinoma-related tumor progression [16]. By contrast, the functional role of PBK/TOPK in ICC remains less well defined. Considering its consistent upregulation, survival association in the TCGA-CHOL cohort, kinase-related biological background, and limited ICC-specific characterization, PBK was selected for focused experimental validation.

### Expression verification of core gene PBK in ICC clinical samples and cell lines

**Fig. 4:**
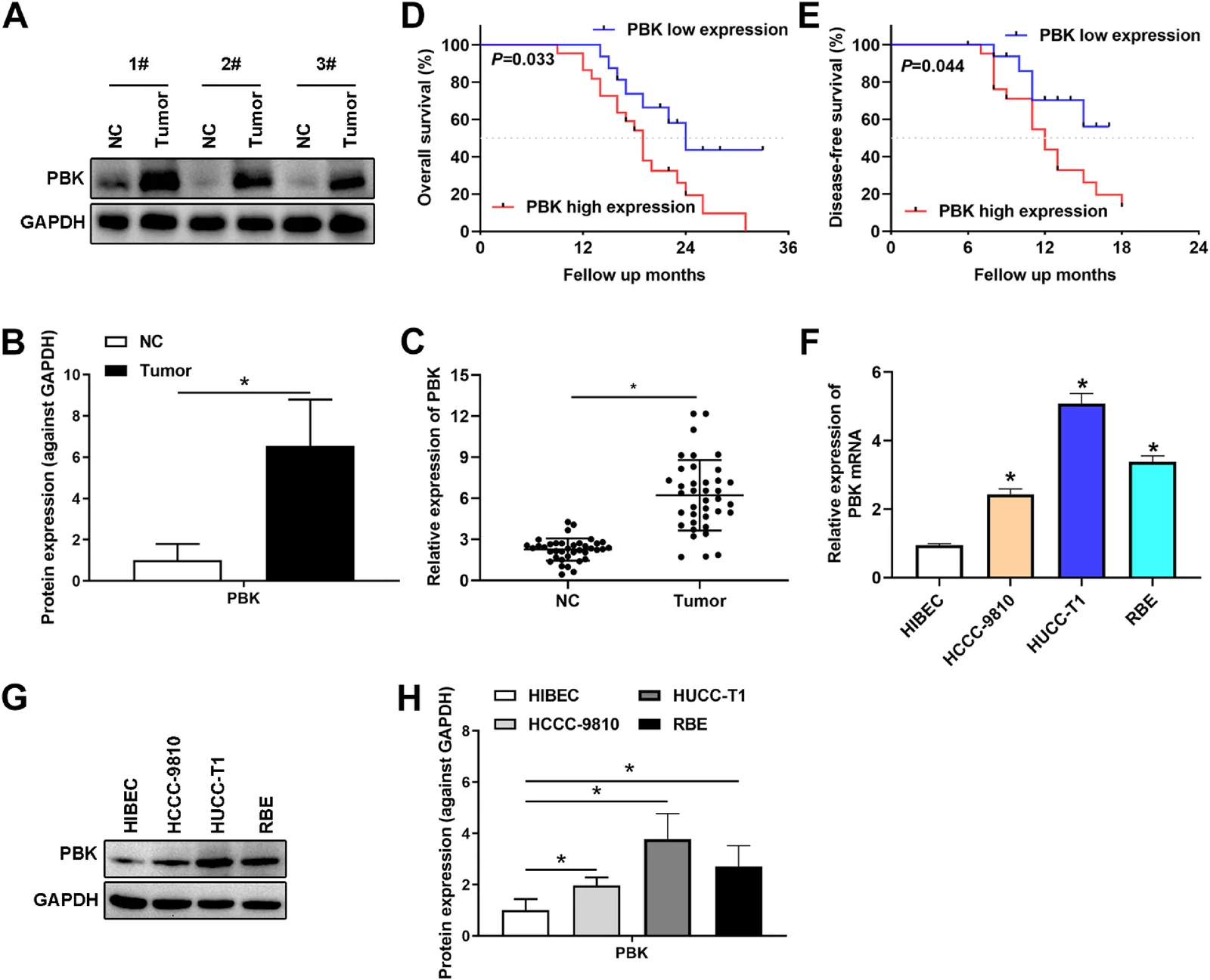
Validation of PBK expression in ICC clinical samples and cell lines. A. PBK protein expression in three paired ICC tissues and adjacent non-tumor tissues, examined by Western blot. B. Densitometric analysis of PBK protein bands. C. RT-qPCR analysis of PBK mRNA expression in ICC tissues and adjacent non-tumor tissues from the separate 38-patient cohort used for clinicopathological and survival analyses. D. Kaplan-Meier curve for overall survival in ICC patients stratified by PBK expression. E. Kaplan-Meier curve for disease-free survival in ICC patients stratified by PBK expression. F. PBK mRNA expression in HIBEC cells and ICC cell lines, including HCCC-9810, HUCC-T1, and RBE, detected by RT-qPCR. G. PBK protein expression in HIBEC, HCCC-9810, HUCC-T1, and RBE cells, assessed by Western blot. H. Densitometric analysis of PBK protein bands. Data are presented as mean ± SD. For cell-line experiments, data were obtained from three independent experiments. *P < 0.05.

Subsequently, we validated PBK expression in ICC clinical samples and cell lines and explored its association with clinicopathological features and patient survival. Western blot assay demonstrated that PBK protein expression was increased in three paired ICC tissue samples compared with adjacent non-tumor tissues (Fig. 4A), and densitometric quantification supported this increase (Fig. 4B). These three paired samples were used only for protein-level validation. In a separate 38-patient cohort, PBK mRNA expression was measured by RT-qPCR and used for clinicopathological correlation and survival analyses. Patients were stratified into PBK-high and PBK-low groups according to the tumor-to-adjacent non-tumor expression ratio, using 2.51 as the cutoff value. Correlation analysis of PBK expression status and clinicopathological features is presented in Table 1. PBK expression was associated with TNM stage and selected clinicopathological variables listed in Table 1. RT-qPCR showed higher PBK mRNA expression in ICC tissues than in adjacent non-tumor tissues in this cohort (Fig. 4C). Kaplan-Meier survival curves showed that patients with higher PBK expression had shorter OS and DFS (Fig. 4D and E), indicating that PBK expression was associated with unfavorable survival in this cohort. Next, we examined PBK expression in different ICC cell lines (HCCC-9810, HUCC-T1, RBE) using RT-qPCR. Consistent with prior observations, PBK expression was markedly elevated in all ICC cell lines relative to HIBEC (Fig. 4F). Western blot further validated elevated expression of PBK in ICC cell lines. In line with RT-qPCR results, Western blot assay demonstrated PBK protein expression levels were remarkably elevated in ICC cell lines than in HIBEC (Fig. 4G and H).

**Table 1:** Relationship between PBK expression and clinicopathologic parameters of ICC patients.

Characteristics	Total	High expression (*n* = 22)	Low expression (*n* = 16)	*P*-value
Age (year)				
≤55	14	8	6	0.943
>55	24	14	10	
Sex				
Female	17	10	7	0.917
Male	21	12	9	
CA19-9				
≤35	11	6	5	0.790
>35	27	16	11	
Pathological				
Well or moderately	17	6	11	0.011*
Poorly	21	16	5	
Vascular invasion				
No	16	6	10	0.030*
Yes	22	16	6	
Tumor Number				
Single	19	8	11	0.049*
Multiple	19	14	5	
Tumor Size				
≤5	14	5	9	0.034*
>5	24	17	7	
T-Stage				
T1	18	9	9	0.350
T2 or T3	20	13	7	
Lymphatic metastasis				
N0	19	8	11	0.049*
N1	19	14	5	
TNM-Stage				
I or II	16	6	10	0.030*
III or IV	22	16	6	

Abbreviations: T stage, tumor stage; TNM, tumor node metastasis.

### Effects of PBK knockdown on ICC cell proliferation, migration, and invasion

**Fig. 5:**
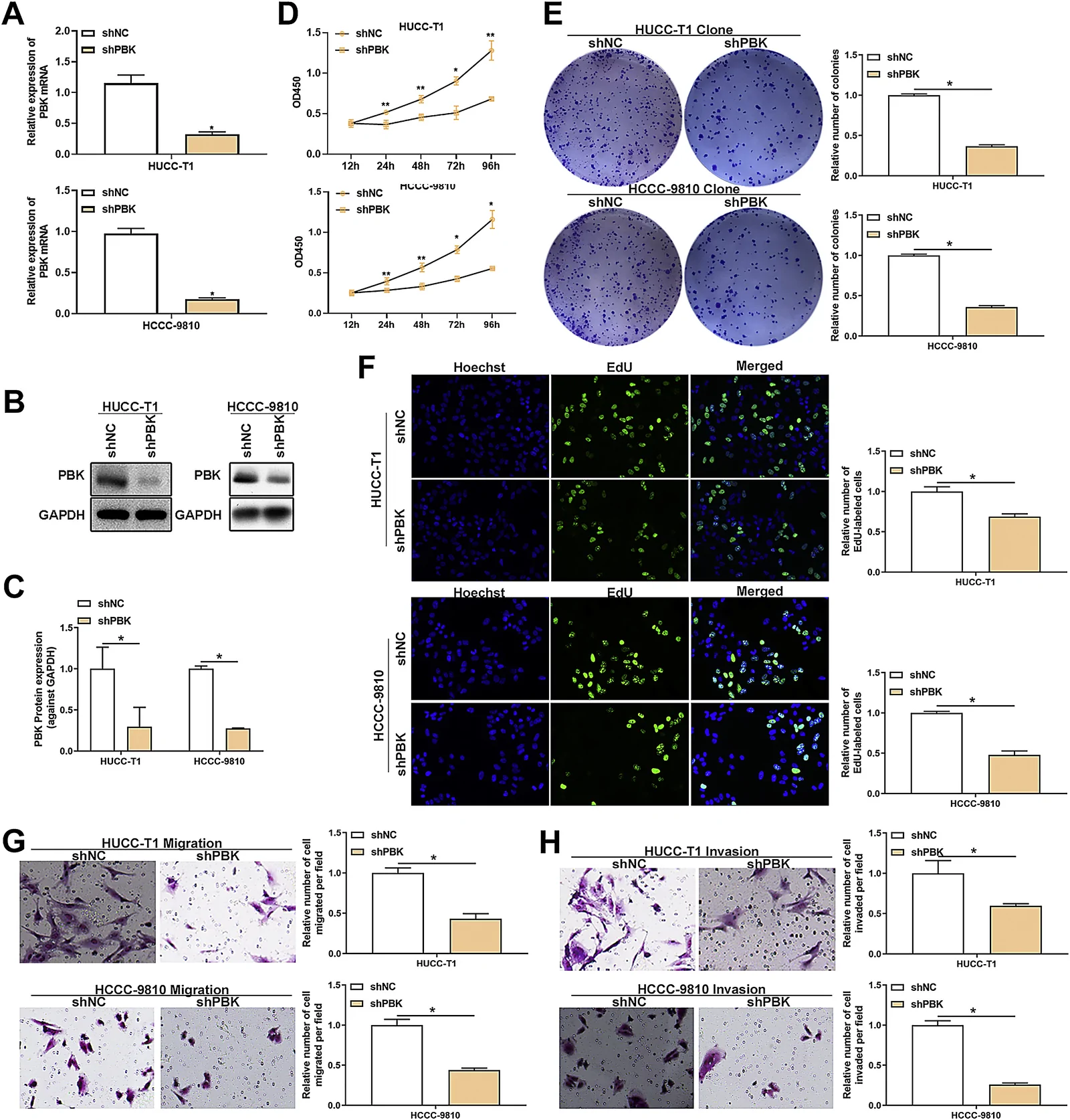
Effects of PBK knockdown on ICC cell proliferation, migration, and invasion. Stable PBK-knockdown models were established in HUCC-T1 and HCCC-9810 cells, with shNC cells used as controls. A. RT-qPCR analysis of PBK mRNA expression in HUCC-T1-shNC, HUCC-T1-shPBK, HCCC-9810-shNC, and HCCC-9810-shPBK cells. B. Western blot detection of PBK protein expression in the indicated cells. C. Densitometric analysis of PBK protein bands. D. CCK-8 assay measuring cell growth at 12, 24, 48, 72, and 96 h. E. Colony formation assay performed after 7 days of culture. F. EdU incorporation assay performed at 48 h. G. Transwell assay evaluating cell migration at 72 h. H. Transwell assay evaluating cell invasion at 72 h. Data are presented as mean ± SD from three independent experiments. *P < 0.05.

To investigate the impacts of PBK knockdown on ICC cell proliferation, migration, and invasion, we established stable PBK-knockdown HUCC-T1 and HCCC-9810 cell lines, which were divided into shNC and shPBK groups for subsequent functional assays. RT-qPCR and Western blot assays confirmed that, compared with the shNC group, PBK mRNA and protein expression were downregulated in the shPBK group (Fig. 5A–C). Functional assays demonstrated that PBK knockdown reduced ICC cell proliferation, as reflected by decreased cell viability, reduced colony-forming ability after 7 days of culture, and fewer EdU-positive cells (Fig. 5D–F). In addition, the numbers of migrated and invaded cells were decreased in the shPBK group relative to the shNC group (Fig. 5G and H). These results indicated that PBK knockdown reduced the proliferative, migratory, and invasive capacities of ICC cells in both HUCC-T1 and HCCC-9810 cell models.

### Effects of PBK overexpression on ICC cell proliferation, migration, and invasion

**Fig. 6:**
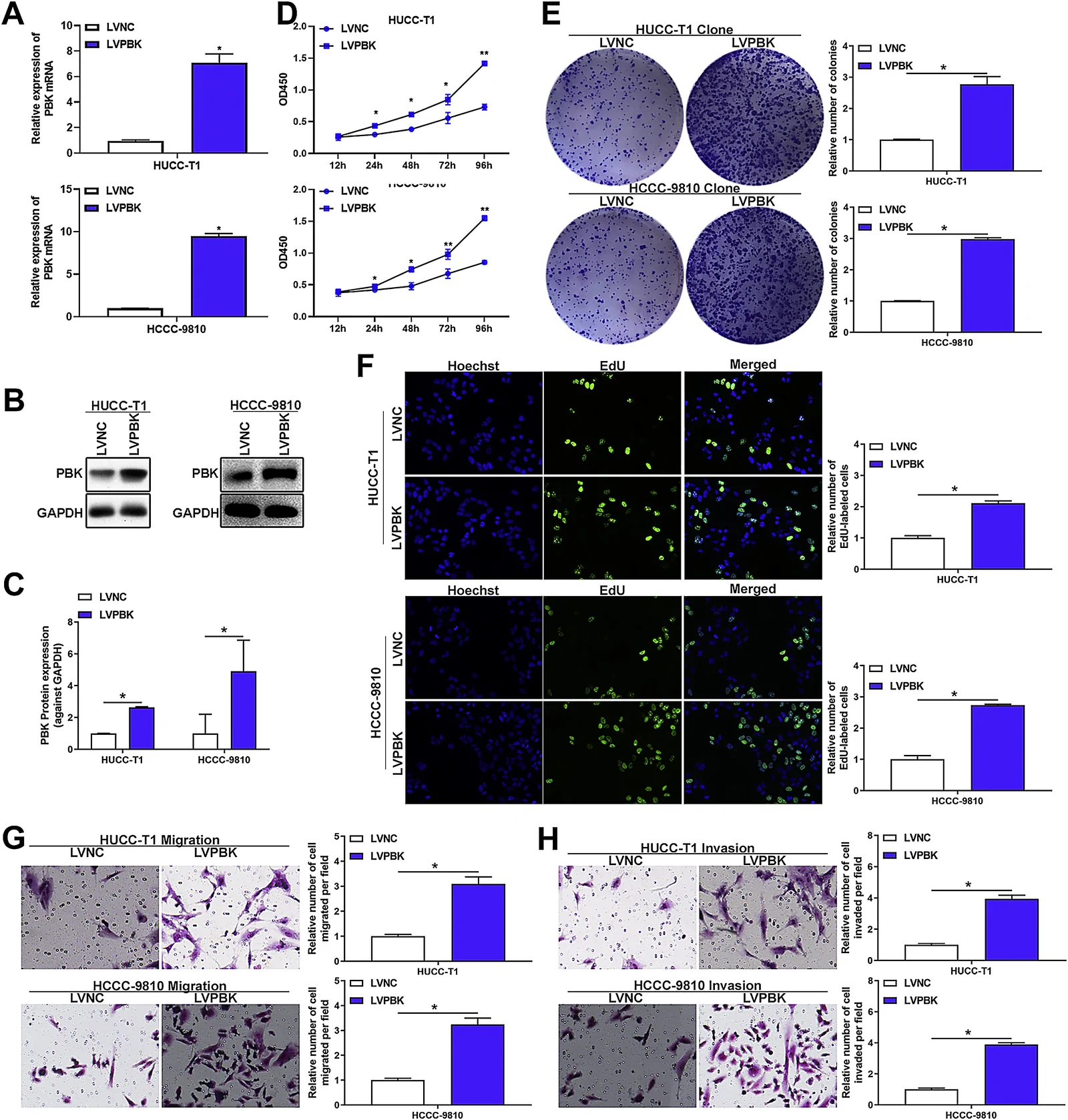
Effects of PBK overexpression on ICC cell proliferation, migration, and invasion. Stable PBK-overexpressing models were generated in HUCC-T1 and HCCC-9810 cells, with LVNC cells used as controls. A. RT-qPCR analysis of PBK mRNA expression in HUCC-T1-LVNC, HUCC-T1-LVPBK, HCCC-9810-LVNC, and HCCC-9810-LVPBK cells. B. Western blot detection of PBK protein expression in the indicated cells. C. Densitometric analysis of PBK protein level. D. CCK-8 assay measuring cell growth at 12, 24, 48, 72, and 96 h. E. Colony formation assay performed after 7 days of culture. F. EdU incorporation assay performed at 48 h. G. Transwell assay assessing cell migration at 72 h. H. Transwell assay assessing cell invasion at 72 h. Data are presented as mean ± SD from three independent experiments. *P < 0.05, **P < 0.01.

To explore the impacts of PBK overexpression on ICC cell proliferation, migration, and invasion, we established stable PBK-overexpressing HUCC-T1 and HCCC-9810 cell lines, which were divided into LVNC and LVPBK groups for subsequent functional assays. Compared with the LVNC group, PBK mRNA and protein expression were elevated in the LVPBK group, confirming PBK overexpression (Fig. 6A–C). Functional assays showed that PBK overexpression increased ICC cell proliferation, as reflected by higher cell viability, enhanced colony-forming ability after 7 days of culture, and an increased proportion of EdU-positive cells in the LVPBK group (Fig. 6D–F). Furthermore, the migratory and invasive capacities of ICC cells were enhanced in the LVPBK group relative to the LVNC group (Fig. 6G and H). These data showed that PBK overexpression promoted proliferation, migration, and invasion in both HUCC-T1 and HCCC-9810 cells.

### AKT signaling was involved in PBK-regulated ICC cell phenotypes

**Fig. 7:**
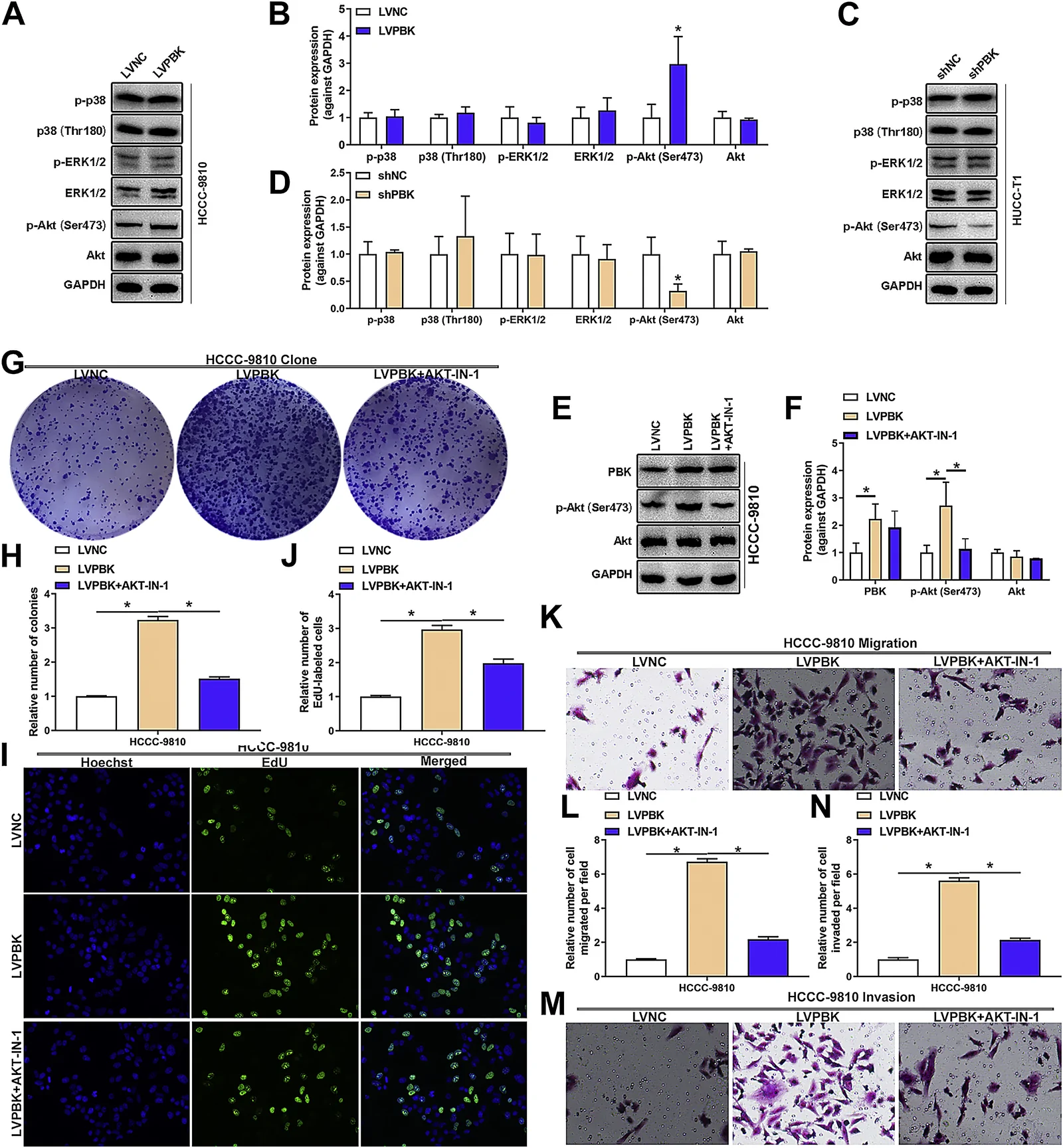
Involvement of AKT signaling in PBK-regulated ICC cell phenotypes. A. Western blot analysis of p-p38 (Thr180), p38, p-ERK1/2, ERK1/2, p-AKT (Ser473), and AKT in HCCC-9810-LVNC and HCCC-9810-LVPBK cells. B. Densitometric analysis of the protein bands in panel A. C. Western blot analysis of p-p38 (Thr180), p38, p-ERK1/2, ERK1/2, p-AKT (Ser473), and AKT in HUCC-T1-shNC and HUCC-T1-shPBK cells. D. Densitometric analysis of the protein bands in panel C. E. Western blot analysis of PBK, p-AKT (Ser473), and AKT in HCCC-9810-LVNC, HCCC-9810-LVPBK, and HCCC-9810-LVPBK + AKT-IN-1 cells. F. Densitometric analysis of the protein bands in panel E. G. Colony formation assay performed after 7 days of culture in HCCC-9810-LVNC, HCCC-9810-LVPBK, and HCCC-9810-LVPBK + AKT-IN-1 cells. H. Quantification of colony numbers. I. EdU incorporation assay performed at 48 h. J. Quantification of EdU-positive cells. K. Transwell migration assay performed at 72 h. L. Quantification of migrated cells. M. Transwell invasion assay performed at 72 h. N. Quantification of invaded cells. AKT-IN-1 was used at 1 μM. Data are presented as mean ± SD from three independent experiments. *P < 0.05.

To further explore signaling changes associated with PBK function, we examined MAPK- and AKT-related proteins by Western blot. In HCCC-9810 cells, PBK overexpression increased the phosphorylation level of AKT at Ser473, while total AKT expression remained relatively unchanged (Fig. 7A and B). By contrast, PBK knockdown reduced p-AKT (Ser473) expression without an obvious change in total AKT (Fig. 7C and D). Compared with p-AKT, changes in p-p38 and p-ERK1/2 were less evident under PBK modulation. These results indicated that AKT signaling was associated with PBK-regulated phenotypes in ICC cells. To evaluate the working concentration of AKT-IN-1, HCCC-9810 cells were treated with 0, 0.1, 0.3, 1, or 3 μM AKT-IN-1 for 48 h. CCK-8 analysis showed that 1 μM AKT-IN-1 did not significantly reduce cell viability, whereas 3 μM markedly decreased cell viability (Supplementary Figure S2A). Therefore, 1 μM was selected for subsequent rescue experiments. Western blot analysis further showed that 1 μM AKT-IN-1 reduced p-AKT (Ser473), while p-ERK1/2 and p-p38 (Thr180) were not obviously changed (Supplementary Figure S2B-S2H). We next used AKT-IN-1 to examine whether AKT inhibition could affect PBK-driven malignant phenotypes. Stable PBK-overexpressing HCCC-9810 cells were divided into three groups: LVNC, LVPBK, and LVPBK + AKT-IN-1. Western blot results showed that PBK overexpression increased p-AKT (Ser473), whereas AKT-IN-1 treatment reduced this increase (Fig. 7E and F). Functionally, PBK overexpression enhanced colony formation, EdU incorporation, migration, and invasion compared with the LVNC group. After AKT-IN-1 treatment, these effects were attenuated, as reflected by reduced colony formation (Fig. 7G and H), fewer EdU-positive cells (Fig. 7I and J), and decreased migratory and invasive capacities (Fig. 7K–N). Together, these results supported the involvement of AKT signaling in PBK-mediated proliferation, migration, and invasion of ICC cells.

### PBK knockdown reduced ICC xenograft growth and AKT-related protein expression in vivo

**Fig. 8:**
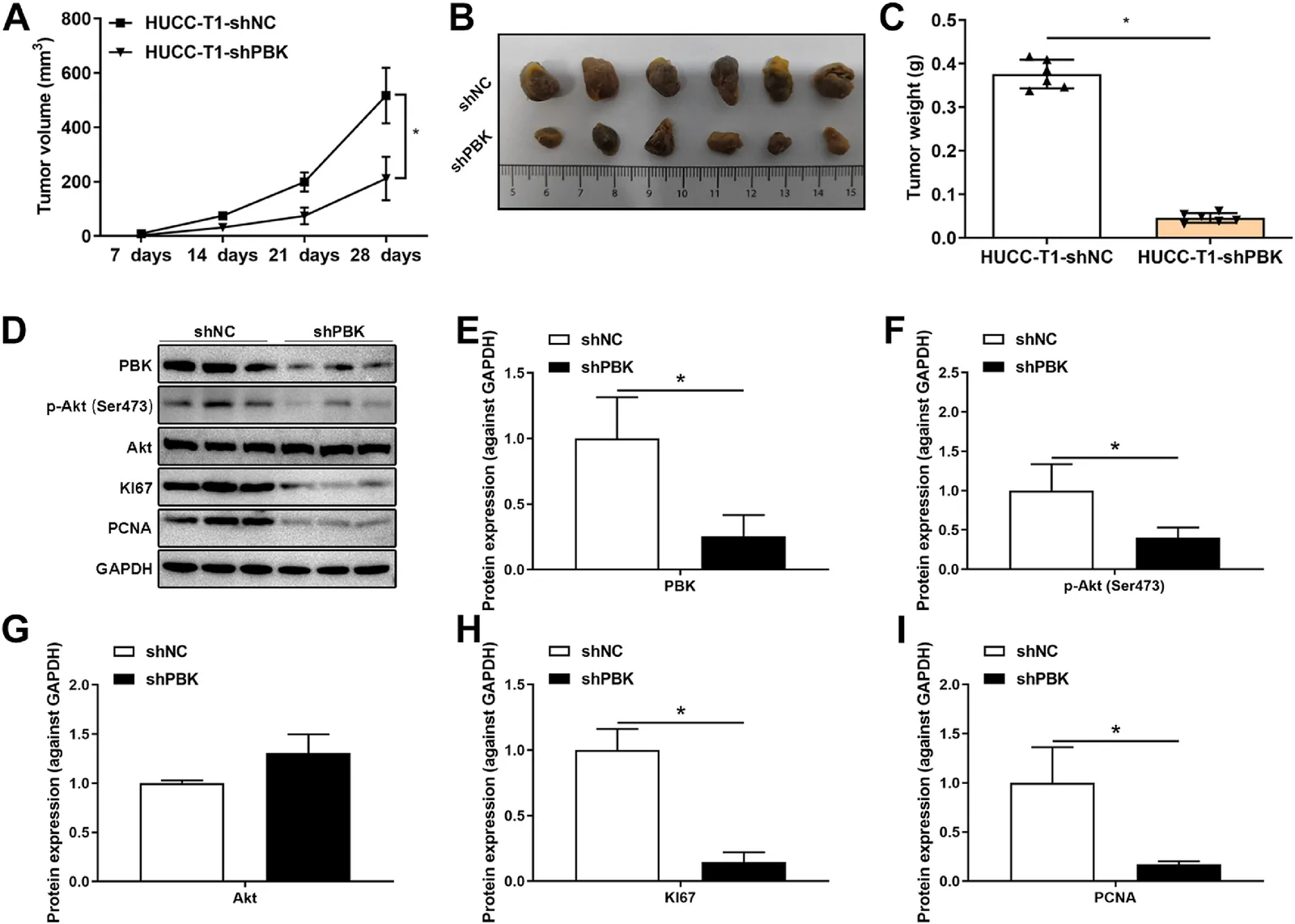
Effects of PBK knockdown on ICC xenograft growth and AKT-related protein expression in vivo. A subcutaneous xenograft model was established by injecting HUCC-T1-shNC or HUCC-T1-shPBK cells into nude mice, with six mice in each group. A. Tumor volume recorded on days 7, 14, 21, and 28 after cell injection. B. Representative images of dissected xenograft tumors. C. Tumor weight measured after tumor collection. D. Western blot analysis of PBK, p-AKT (Ser473), AKT, KI67, and PCNA expression in xenograft tumor tissues. E-I. Densitometric analysis of protein levels in panel D. Data are presented as mean ± SD. *n* = 6 mice per group. *P < 0.05.

Finally, we examined the effect of PBK knockdown on ICC tumor growth in vivo using a subcutaneous xenograft model in nude mice. HUCC-T1-shNC and HUCC-T1-shPBK cells were injected subcutaneously, and tumor growth was monitored over time. Compared with the HUCC-T1-shNC group, tumor growth was slower in the HUCC-T1-shPBK group (Fig. 8A). Representative tumor images and tumor weight analysis further showed reduced xenograft growth after PBK knockdown (Fig. 8B and C). Western blot assay demonstrated that PBK knockdown decreased PBK protein expression in xenograft tissues. In parallel, p-AKT (Ser473), KI67, and PCNA levels were reduced in the HUCC-T1-shPBK group, whereas total AKT showed no marked change (Fig. 8D–I). These in vivo results indicated that PBK knockdown inhibited xenograft tumor growth and was accompanied by reduced AKT phosphorylation and lower expression of proliferation-related proteins.

## Discussion

The high invasiveness and poor prognosis of ICC have driven continuous efforts to uncover its underlying molecular drivers [17]. In this study, we identified and functionally evaluated PBK as a candidate molecule associated with ICC progression by integrating multiple bioinformatics analysis strategies using data from TCGA. Combined with clinical sample validation, in vitro functional assays, pathway-related analyses, and an in vivo xenograft model, our results suggest that PBK contributes to malignant phenotypes of ICC cells, with AKT signaling involved in this process.

ICC remains a highly invasive malignancy with limited molecular indicators for risk assessment and limited targeted therapeutic options for many patients [18]. In this study, we analyzed the TCGA-CHOL dataset using three tools—DESeq2, edgeR, and limma—to identify differentially expressed genes in CHOL. Combined with WGCNA and PPI network screening, PBK was prioritized as a candidate gene for further validation. Clinical validation showed that PBK was elevated in ICC tissues and cells, and its expression was associated with TNM stage and unfavorable survival outcomes in the clinical cohort. ROC curve analysis in the TCGA-CHOL cohort suggested potential discriminatory performance of PBK in this internal discovery dataset; however, this finding requires validation in independent external cohorts before any diagnostic conclusion can be made. This observation is generally consistent with previous studies on PBK in other malignancies, such as glioblastoma and breast cancer [19,20]. PBK, also known as TOPK, is a serine/threonine kinase of the MAPKK family and has been implicated in cell-cycle progression, cell polarity modulation, and cell migratory capacity [13,21,22]. Accumulating evidence has shown that PBK is dysregulated in various tumor types, such as breast, prostate, and colorectal cancers, and may participate in tumorigenesis and progression by regulating cell proliferation, apoptosis, invasion, and metastasis [20,23,24]. Although PBK has been investigated in several malignancies, its functional role in ICC progression and its relationship with AKT signaling remain insufficiently characterized. In the present study, PBK was selected for experimental validation based on its upregulation, survival association, kinase-related biological background, and limited functional characterization in ICC. NEK2 also showed bioinformatic relevance in the TCGA-CHOL cohort; however, NEK2 has already been implicated in cholangiocarcinoma-related tumor growth and dissemination [16]. PBK/TOPK, in contrast, remains less clearly characterized in ICC. The present work therefore focused on PBK to provide a coherent functional evaluation of this candidate kinase in ICC models. Other candidate genes identified in the screening process may also be biologically relevant and deserve separate investigation.

To investigate the biological functions of PBK, we conducted gain-of-function and loss-of-function experiments in ICC cells. Knockdown of PBK expression inhibited ICC cell proliferation, migration, and invasion, whereas PBK overexpression had the opposite effects. These results support a tumor-promoting role of PBK in ICC cell phenotypes. Proliferation, migration, and invasion represent core hallmarks of malignant tumor progression [25], and the regulatory effects of PBK on these processes may be related to its reported functions in cell-cycle regulation, cell polarity, and cytoskeletal remodeling [26]. Therefore, PBK may contribute to ICC progression by promoting cellular behaviors associated with tumor growth and dissemination.

Aberrant activation of signaling pathways is an important feature of tumor progression. The AKT signaling pathway is frequently involved in oncogenic signaling and has been implicated in ICC tumorigenesis and progression. By modulating downstream molecules, AKT signaling regulates multiple biological processes in tumor cells, including proliferation, apoptosis, migration, and angiogenesis [27,28]. Clinical studies have shown that activation of PI3K/AKT/mTOR-related signaling components is associated with malignant clinicopathological features in ICC tissues [29]. In vitro studies have also suggested that inhibition of the PI3K/AKT pathway can suppress ICC cell proliferation and invasion [30]. However, the upstream regulatory network of AKT signaling in ICC remains incompletely understood. In the present study, PBK overexpression increased the phosphorylation level of AKT, whereas PBK knockdown reduced p-AKT expression. Compared with p-AKT, changes in p-ERK1/2 and p-p38 were less evident under PBK modulation. In the AKT-IN-1 concentration assessment, 1 μM AKT-IN-1 reduced p-AKT expression without obvious changes in p-ERK1/2 or p-p38, supporting its use in subsequent rescue assays. Functionally, AKT-IN-1 attenuated PBK-overexpression-induced colony formation, EdU incorporation, migration, and invasion. These findings support the involvement of AKT signaling in PBK-regulated ICC cell phenotypes. Previous studies have reported that PBK/TOPK can regulate cell migration through a PI3K/PTEN/AKT-dependent pathway in lung cancer [31]. Our findings are consistent with this possibility, but the direct molecular link between PBK and AKT activation in ICC remains to be clarified. In vivo, PBK knockdown reduced xenograft tumor growth and was accompanied by decreased p-AKT expression and lower levels of proliferation-related proteins KI67 and PCNA. These results further support the association between PBK expression, AKT phosphorylation, and ICC tumor growth, but additional mechanistic studies are still needed to determine whether PBK directly regulates AKT or acts through upstream mediators such as PTEN or other signaling components.

In recent years, the integration of multi-omics data analysis to identify key driver genes in tumors has become an important strategy in the study of tumor molecular mechanisms [32]. This study combined three differential expression analysis tools with WGCNA, which may reduce the influence of method-specific bias and improve the reliability of candidate gene selection. This approach is consistent with the concept that integration of multiple analytical methods may improve the robustness of biomarker discovery [33,34]. In the field of CHOL research, previous research has used CRISPR library screening to identify key kinase targets such as AURKB, demonstrating that these kinases influence CHOL progression by regulating cholesterol metabolism [35]. PBK, like AURKB, is a kinase gene that was found to be upregulated in ICC and associated with poor prognosis in our analysis. These findings suggest that kinase-related molecules may represent an important group of regulators in ICC progression. Future studies may further compare PBK with other candidate kinases, such as NEK2 and AURKB, to better define their relative contributions and potential interactions in ICC.

The main strength of this study is the integration of bioinformatics screening with clinical sample validation, bidirectional functional experiments, AKT-IN-1 rescue assays, and in vivo validation. This design provides supportive evidence that PBK is involved in ICC progression and that AKT signaling participates in PBK-regulated malignant phenotypes. However, several limitations should be considered. The clinical cohort size was limited, and the association between PBK expression and prognosis requires validation in larger, independent cohorts. In addition, the ROC analysis was based on the TCGA-CHOL discovery dataset and should be regarded as an internal exploratory estimate rather than a validated diagnostic result. Although AKT-IN-1 attenuated PBK-induced malignant phenotypes, the current data do not establish a direct molecular interaction between PBK and AKT. Further studies are needed to examine upstream mediators, such as PTEN, and to clarify how PBK is connected to AKT signaling in ICC. Moreover, the subcutaneous xenograft model does not reproduce the hepatic stromal architecture or immune context of human ICC. We did not perform immune deconvolution analysis to assess the relationship between PBK expression and immune cell populations, including CD8+ T cells, regulatory T cells, and tumor-associated macrophages. Thus, the role of PBK in the ICC immune microenvironment remains unclear. The present data are also insufficient to infer whether PBK-related signaling may influence immunotherapy response or combination treatment strategies. These questions should be addressed in larger transcriptomic cohorts and in orthotopic or immunocompetent models. Overall, the translational implications of PBK-related signaling should be interpreted cautiously and regarded as exploratory at this stage.

## Conclusion

This study identified PBK as a candidate molecule associated with ICC progression through integrated bioinformatics analysis and experimental validation. PBK promoted ICC cell proliferation, migration, and invasion, while PBK knockdown reduced xenograft tumor growth. At the signaling level, our data support the involvement of AKT signaling in PBK-regulated ICC phenotypes, although the direct molecular connection between PBK and AKT remains to be clarified. These findings provide evidence supporting the biological relevance of PBK in ICC and suggest that PBK-related signaling deserves further investigation in future studies.

## Clinical trial number

not applicable.

## Ethics approval statements, informed consent, and consent to participate

Informed written consent was acquired from all participating patients who provided samples, and this study was approved by the Bayannur Hospital Committee (approval number 20251107002, dated November 7, 2025). All procedures performed in studies involving human participants were in accordance with the ethical standards of the institutional and/or national research committee and with the 1964 Helsinki Declaration and its later amendments or comparable ethical standards. All animal experiments were approved by the Guangzhou Forevergen Medical Laboratory Animal Center (approval number: IACUC-AEWC-F251015045, dated October 15, 2025) Committee. Animal experiments were performed in accordance with ARRIVE guidelines and the IACUC Handbook (third edition).

## Funding

This study was supported by the Basic Research Projects of Liaoning Provincial Department of Education (Grant number LJ222510161004) and the Inner Mongolia Natural Science Foundation Youth Project (Grant number 2026QC0015).

## Artificial intelligence statements

No part of this paper was drafted, paraphrased, or ideated by any AI system; an AI tool was consulted solely for minor grammar and spelling corrections.

## CRediT authorship contribution statement

**Xiangtian Shi:** Writing – review & editing, Writing – original draft, Methodology, Investigation, Formal analysis, Data curation, Conceptualization. **Lei Zhang:** Writing – review & editing, Methodology, Investigation, Formal analysis, Data curation, Conceptualization. **Tengqi Wang:** Writing – review & editing, Writing – original draft, Methodology, Investigation, Formal analysis, Data curation, Conceptualization. **Meng Wang:** Writing – review & editing, Writing – original draft, Methodology, Investigation, Formal analysis, Data curation, Conceptualization. **Chao Wang:** Writing – review & editing, Writing – original draft, Methodology, Investigation, Formal analysis, Data curation, Conceptualization. **Zhangxu Liu:** Writing – review & editing, Writing – original draft, Methodology, Investigation, Formal analysis, Data curation, Conceptualization. **Guixin Zhang:** Writing – review & editing, Writing – original draft, Methodology, Investigation, Formal analysis, Data curation, Conceptualization.

## Declaration of competing interest

The authors declare that they have no known competing financial interests or personal relationships that could have appeared to influence the work reported in this paper.

## Data Availability

The original contributions presented in the study are included in the article/supplementary material, further inquiries can be directed to the corresponding author/s.

## Appendix Supplementary materials

**Supplementary Fig. S1. Determination of the soft-thresholding power for WGCNA.** A. Scale-free topology fit index under different soft-thresholding powers. B. Mean connectivity under different soft-thresholding powers. Based on these diagnostic plots, a soft-thresholding power of β = 5 was selected for subsequent WGCNA. Image, image 1 dummy alt text

**Supplementary Fig. S2. Evaluation of AKT-IN-1 working concentration in HCCC-9810 cells.** A. CCK-8 assay of HCCC-9810 cells treated with different concentrations of AKT-IN-1 for 48 h. B. Western blot analysis of p-p38 (Thr180), p38, p-ERK1/2, ERK1/2, p-AKT (Ser473), AKT, and GAPDH in HCCC-9810 cells treated with 1 μM AKT-IN-1 for 48 h. C—H. Densitometric analysis of protein levels in panel B. Data are presented as mean ± SD from three independent experiments. NS, not significant; ***P* < 0.01. Image, image 2 dummy alt text

Table S1. Common key genes identified by intersecting DEGs from TCGA database with hub genes from WGCNA. Image, application 3

Table S2. Results of PPI network analysis conducted on key genes. Image, application 4

Image, application 5
