# Supplementary material for: TCGA-based identification and functional validation of PBK in intrahepatic cholangiocarcinoma progression with involvement of AKT signaling
Source: Transl Oncol. 2026 Aug 13;72:102977. doi: 10.1016/j.tranon.2026.102977 (PMC13495409; doi:10.1016/j.tranon.2026.102977)

Figure 4A

PBK

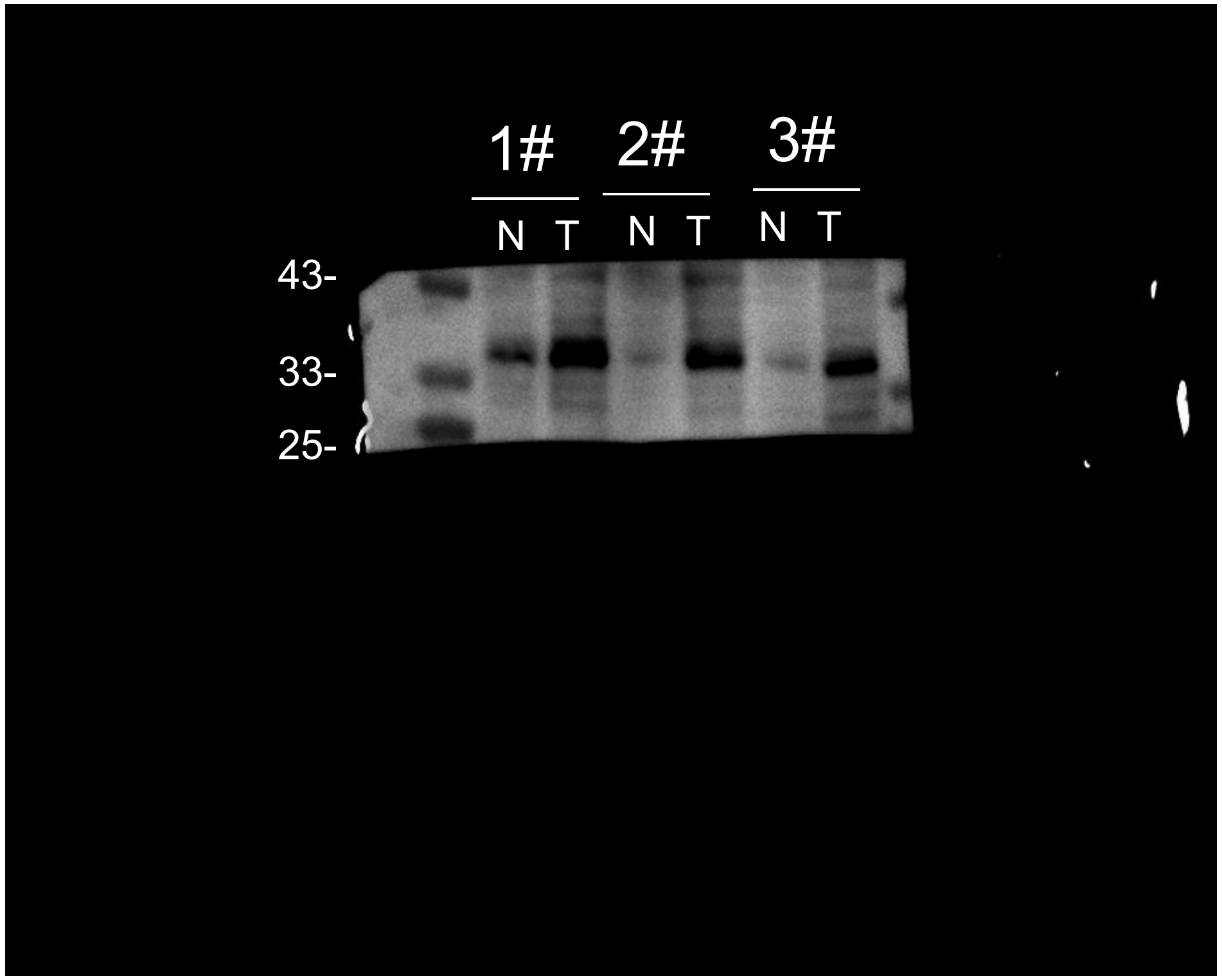

GAPDH

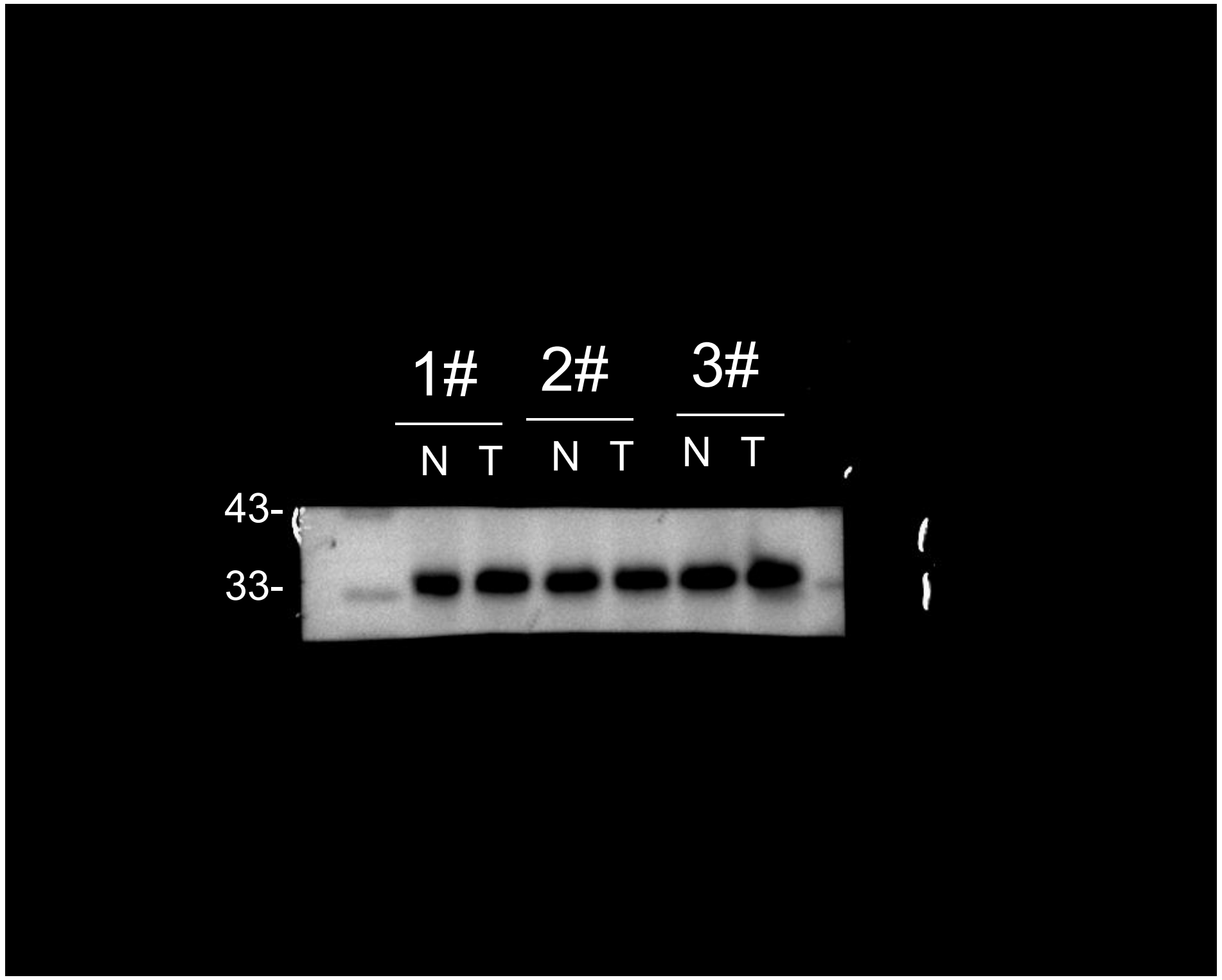

Figure 4F

PBK

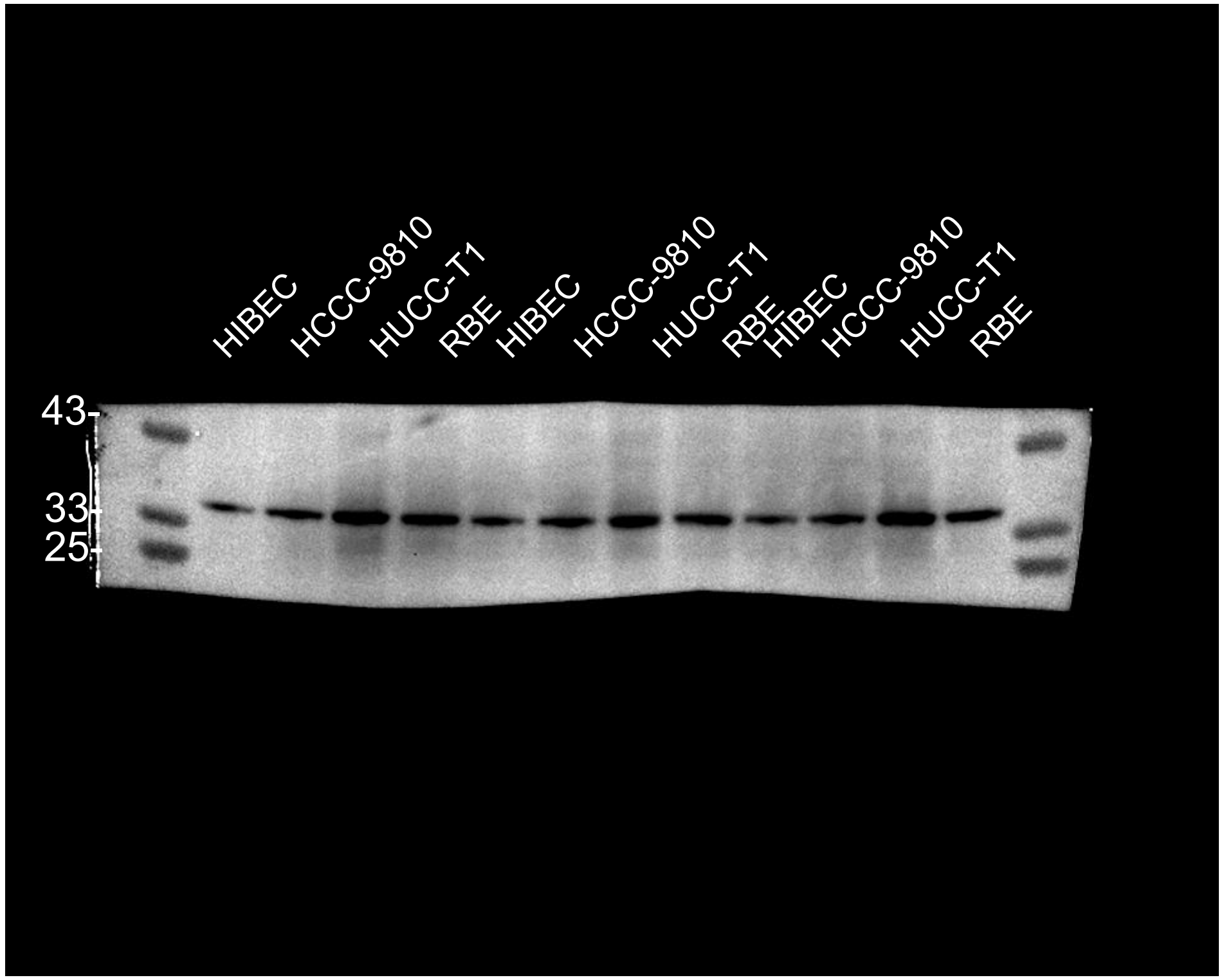

GAPDH

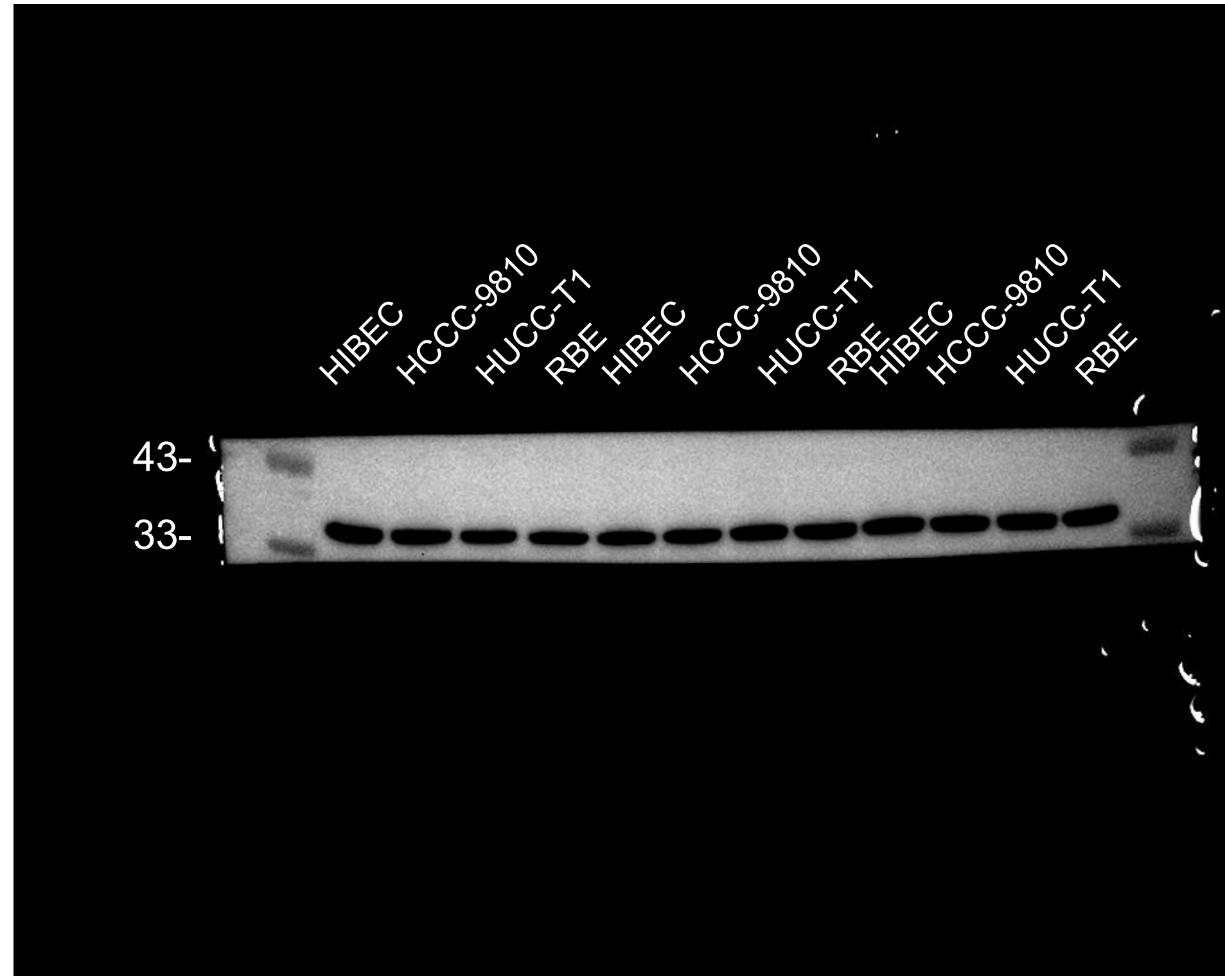

Figure 5B

PBK

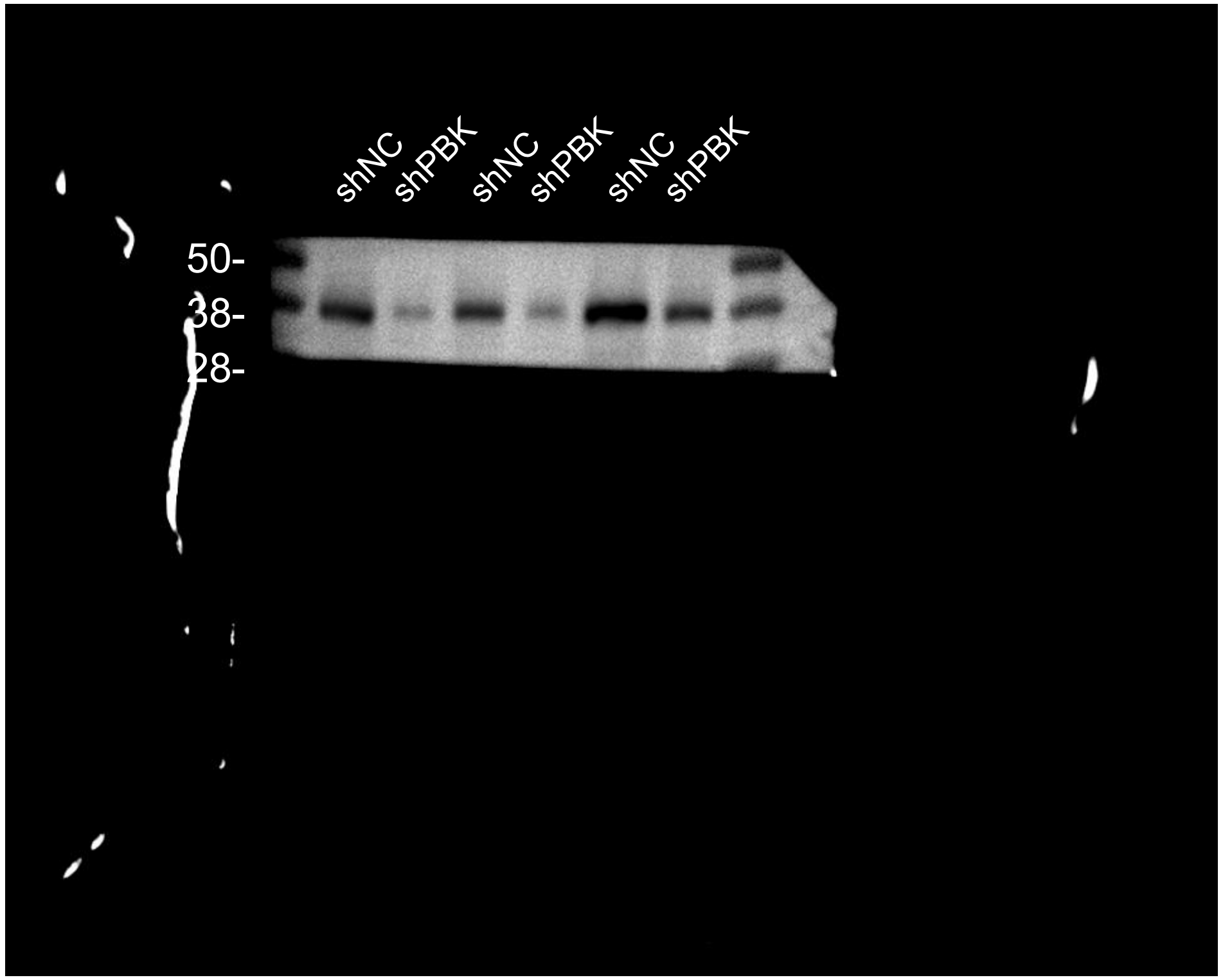

GAPDH

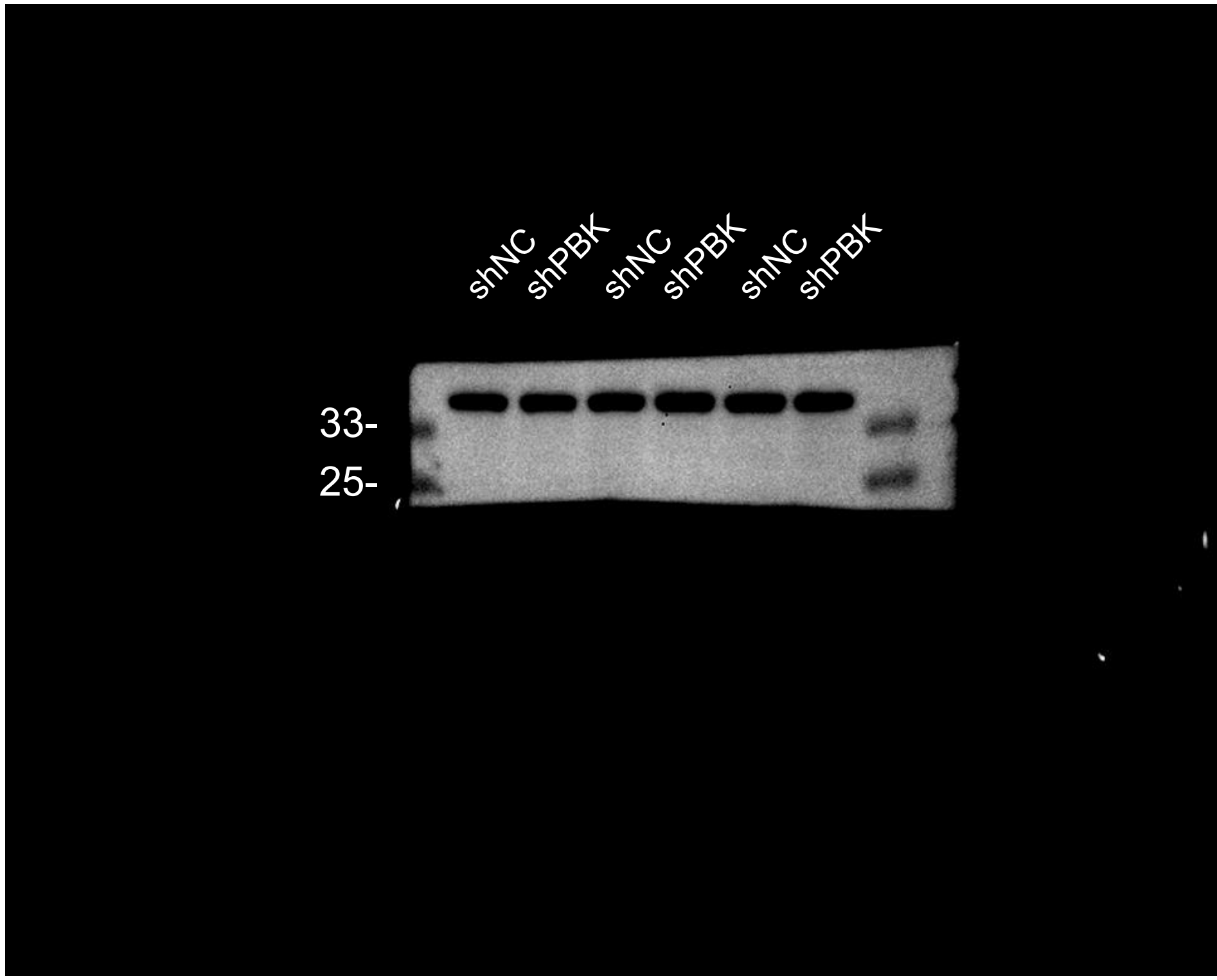

Figure 6B

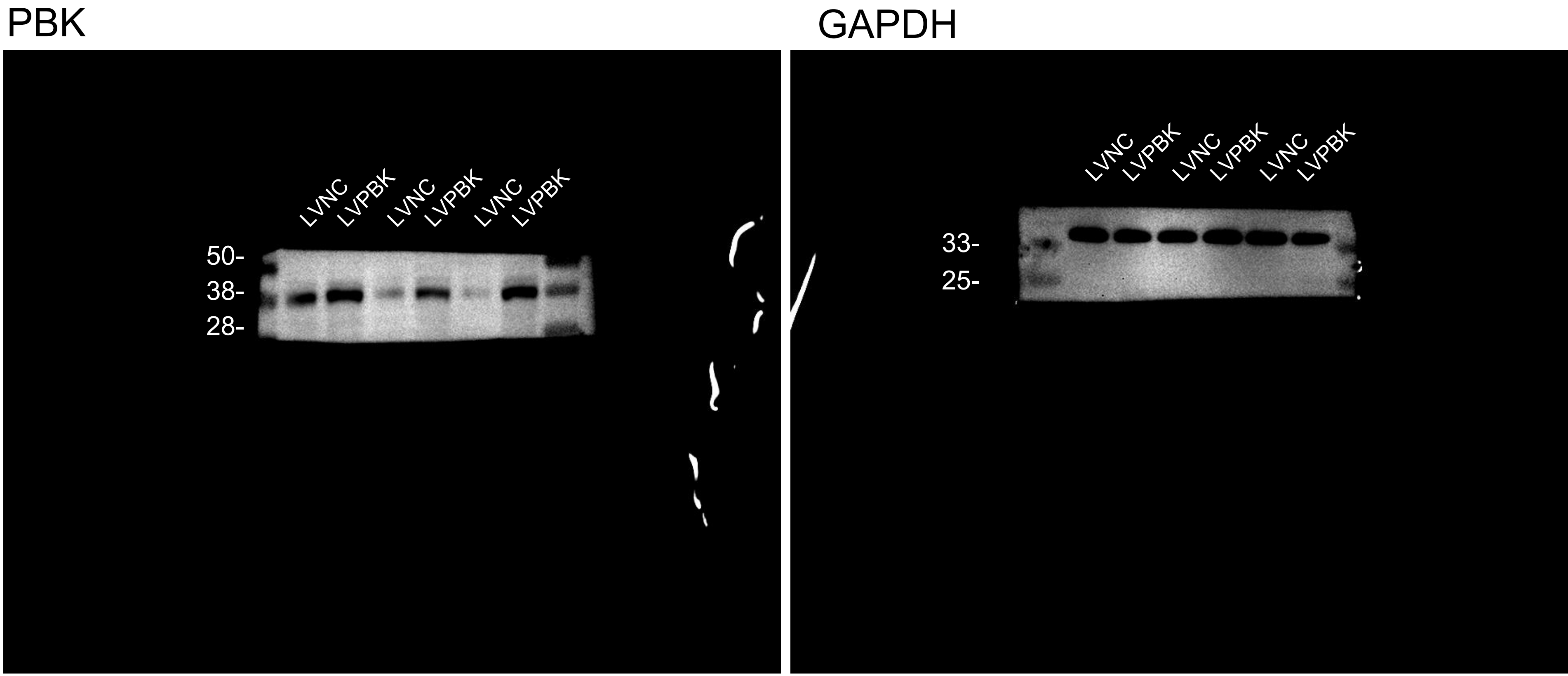
PBK
GAPDH
LVNC
LVPBK
LVNC
LVPBK
LVNC
LVPBK
50-
38-
28-
LVNC
LVPBK
LVNC
LVPBK
LVNC
LVPBK
33-
25-

Figure 7A/B

p-p38

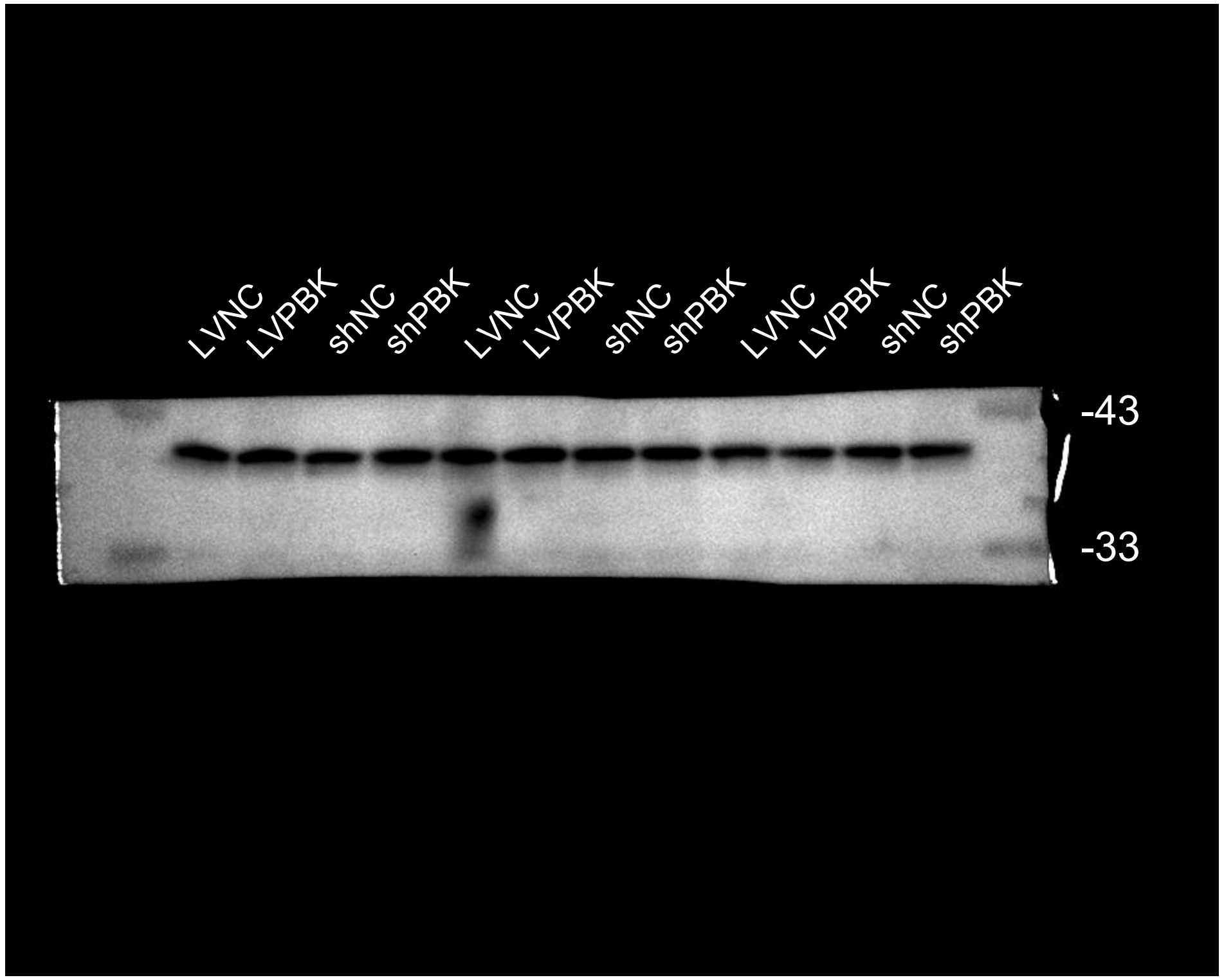

p38

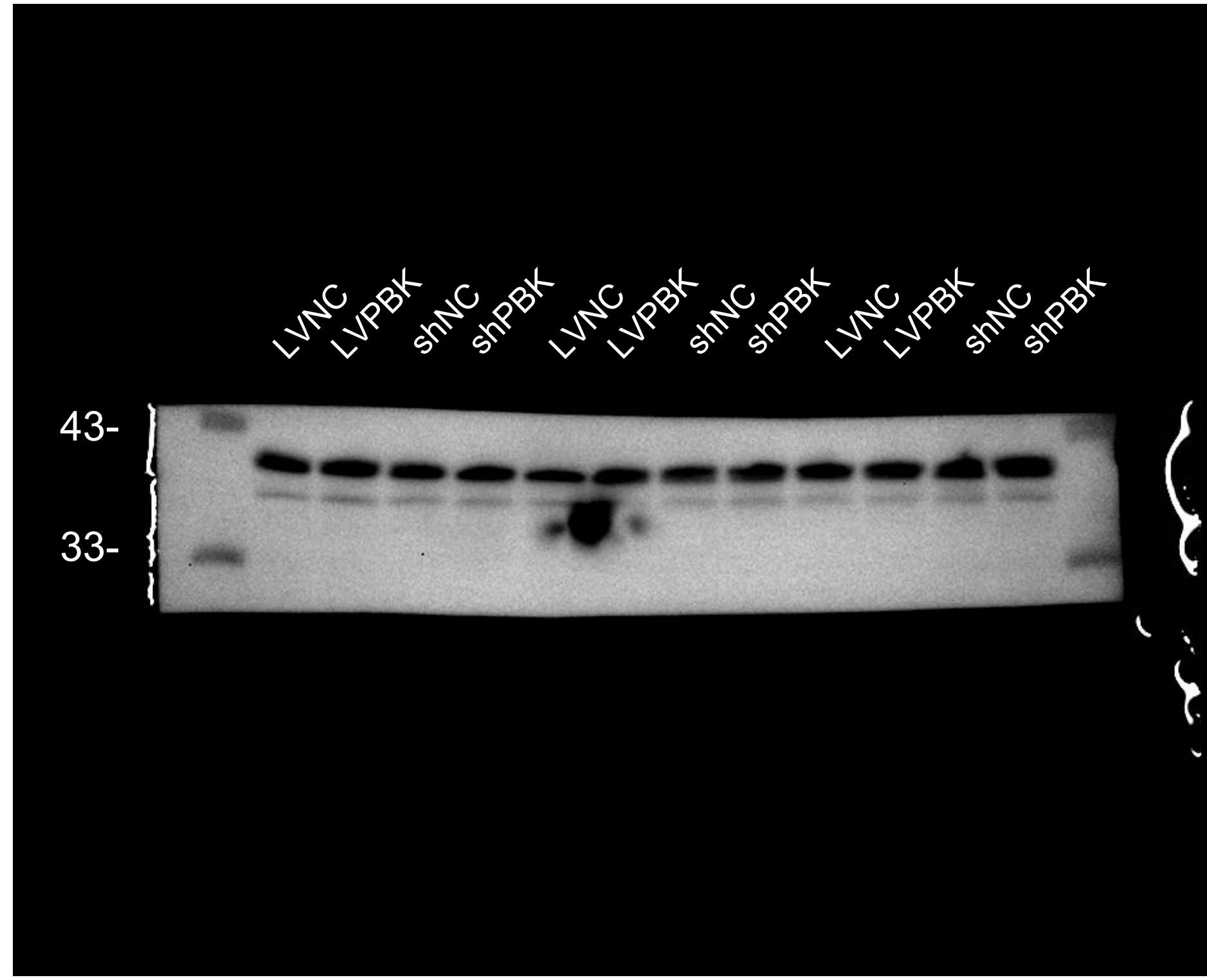

GAPDH

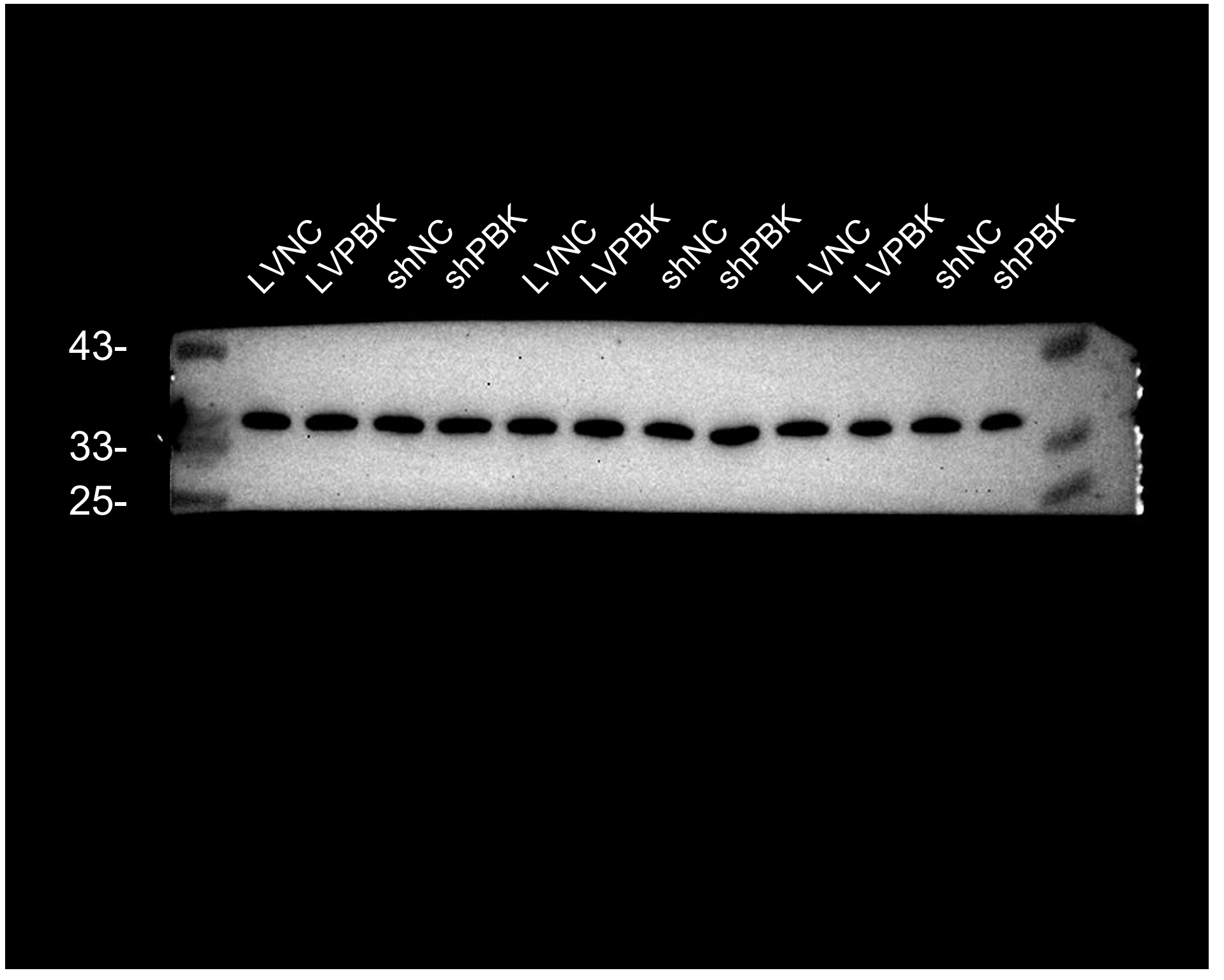

LVNC
LVPBK
shNC
shPBK
LVNC
LVPBK
shNC
shPBK
LVNC
LVPBK
shNC
shPBK
43-
33-
25-

p-ERK1/2

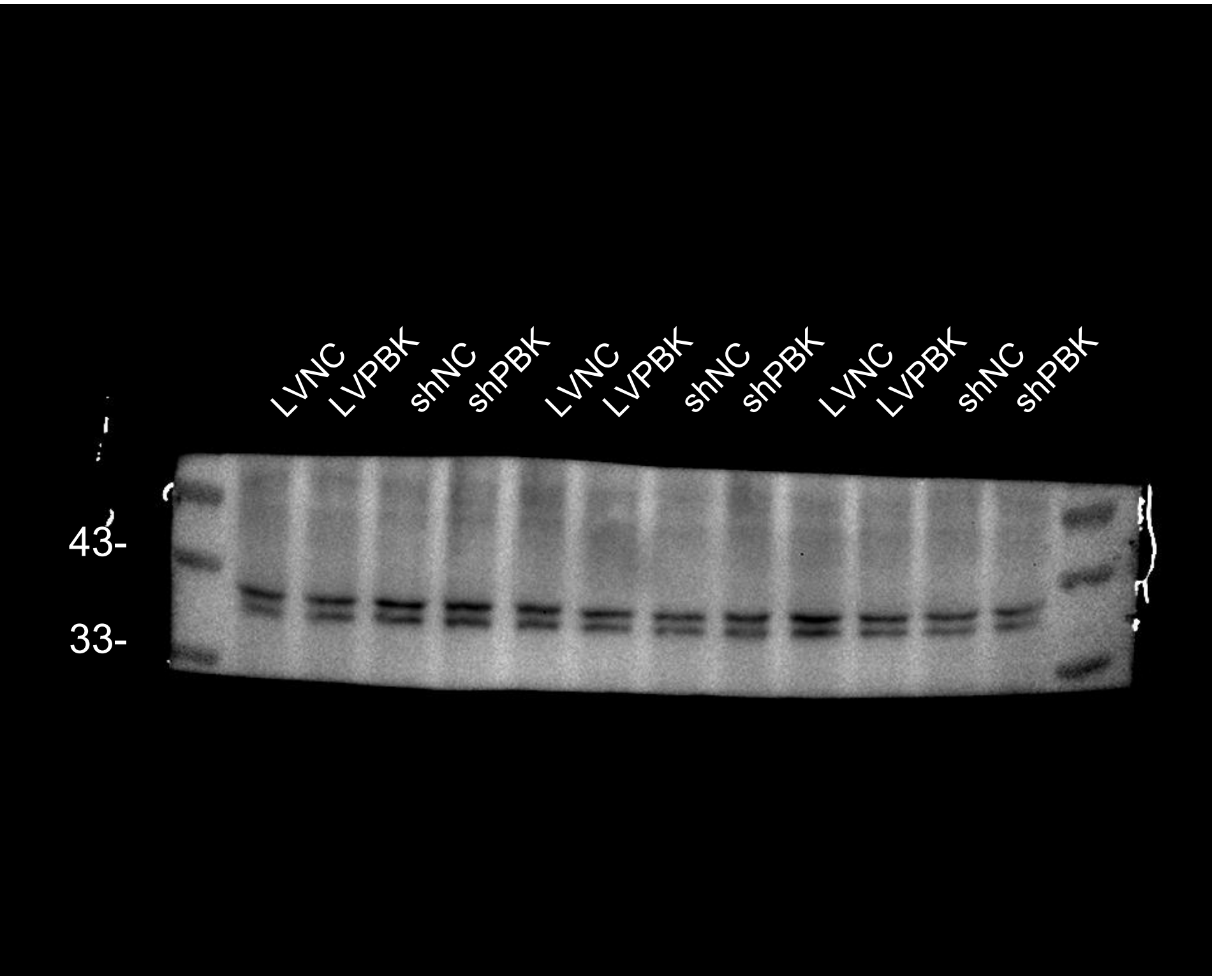
LVNC
LVPBK
shNC
shPBK
LVNC
LVPBK
shNC
shPBK
LVNC
LVPBK
shNC
shPBK
43-
33-

ERK2

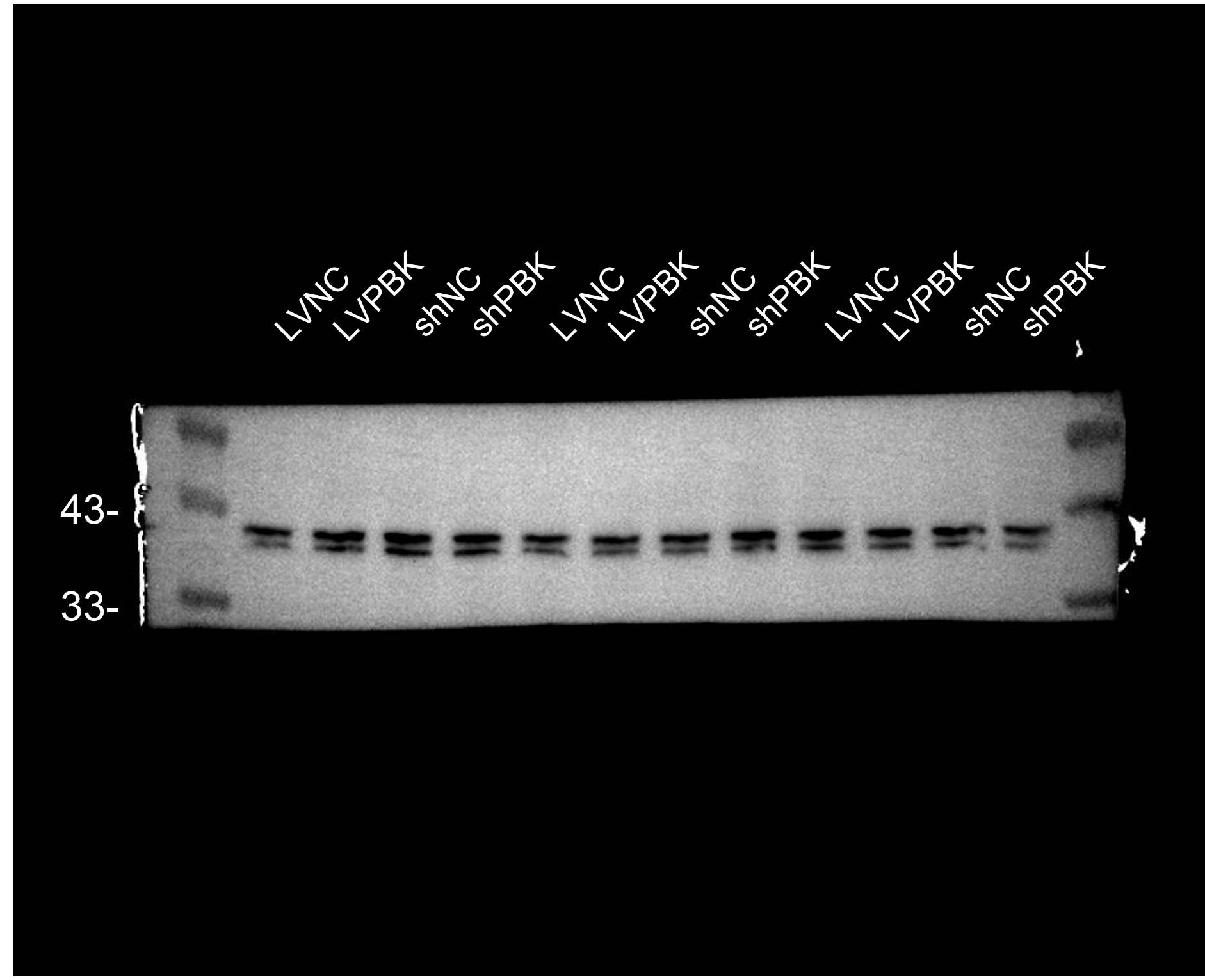
LVNC
LVPBK
shNC
shPBK
LVNC
LVPBK
shNC
shPBK
LVNC
LVPBK
shNC
shPBK
43-
33-

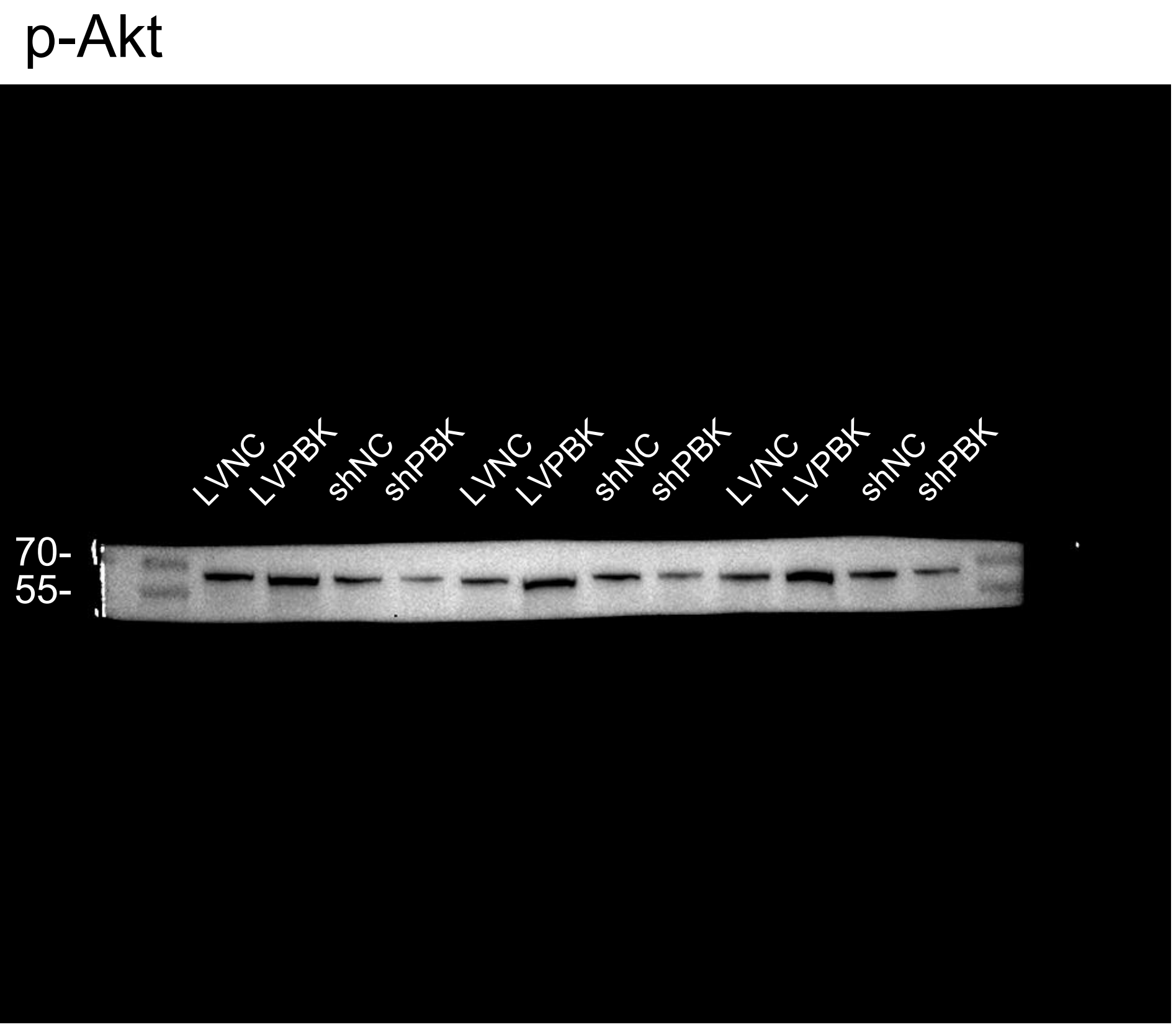
p-Akt
LVNC
LVPBK
shNC
shPBK
LVNC
LVPBK
shNC
shPBK
LVNC
LVPBK
shNC
shPBK
70-
55-
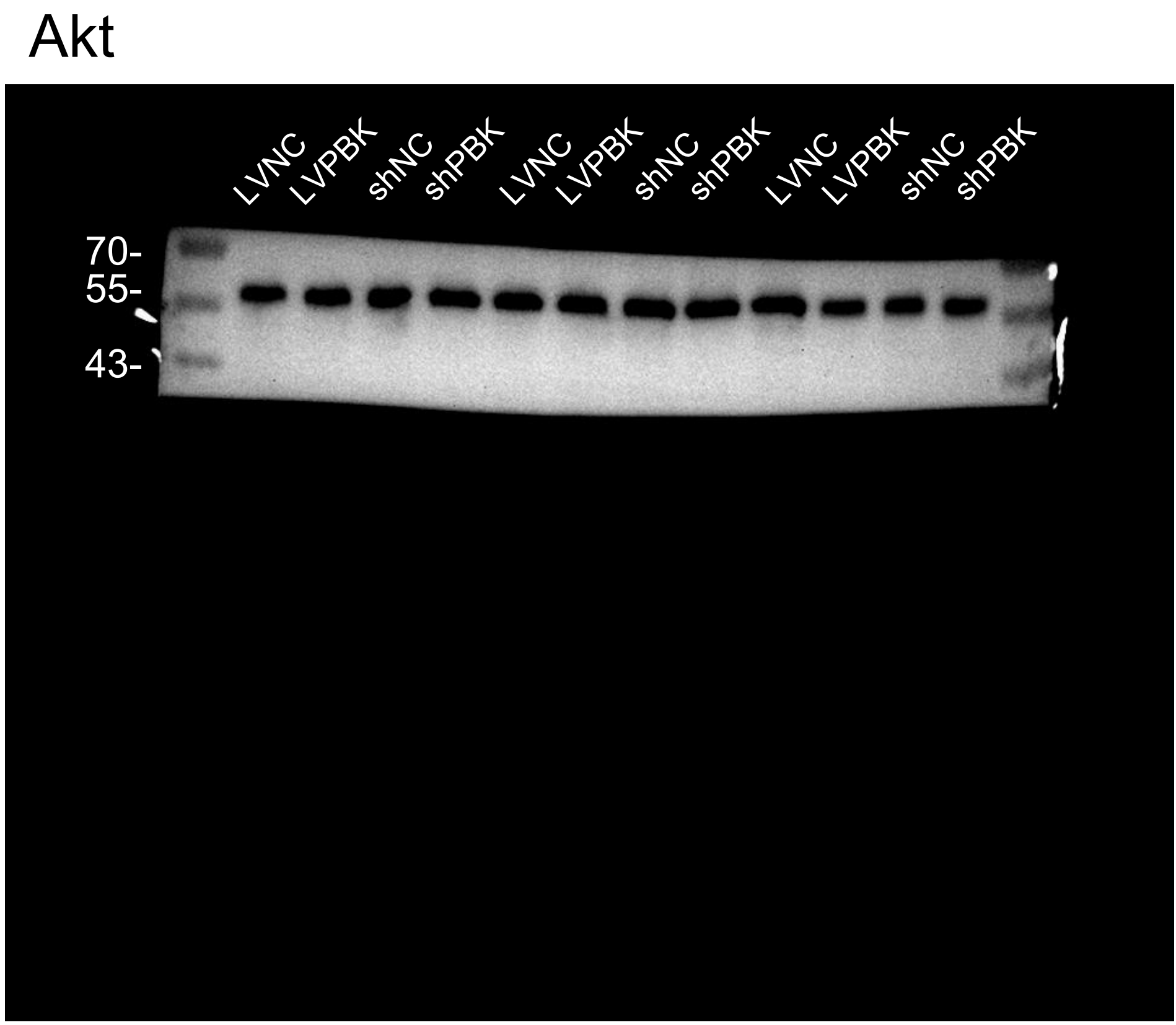
Akt
LVNC
LVPBK
shNC
shPBK
LVNC
LVPBK
shNC
shPBK
LVNC
LVPBK
shNC
shPBK
70-
55-
43-

Figure 7C

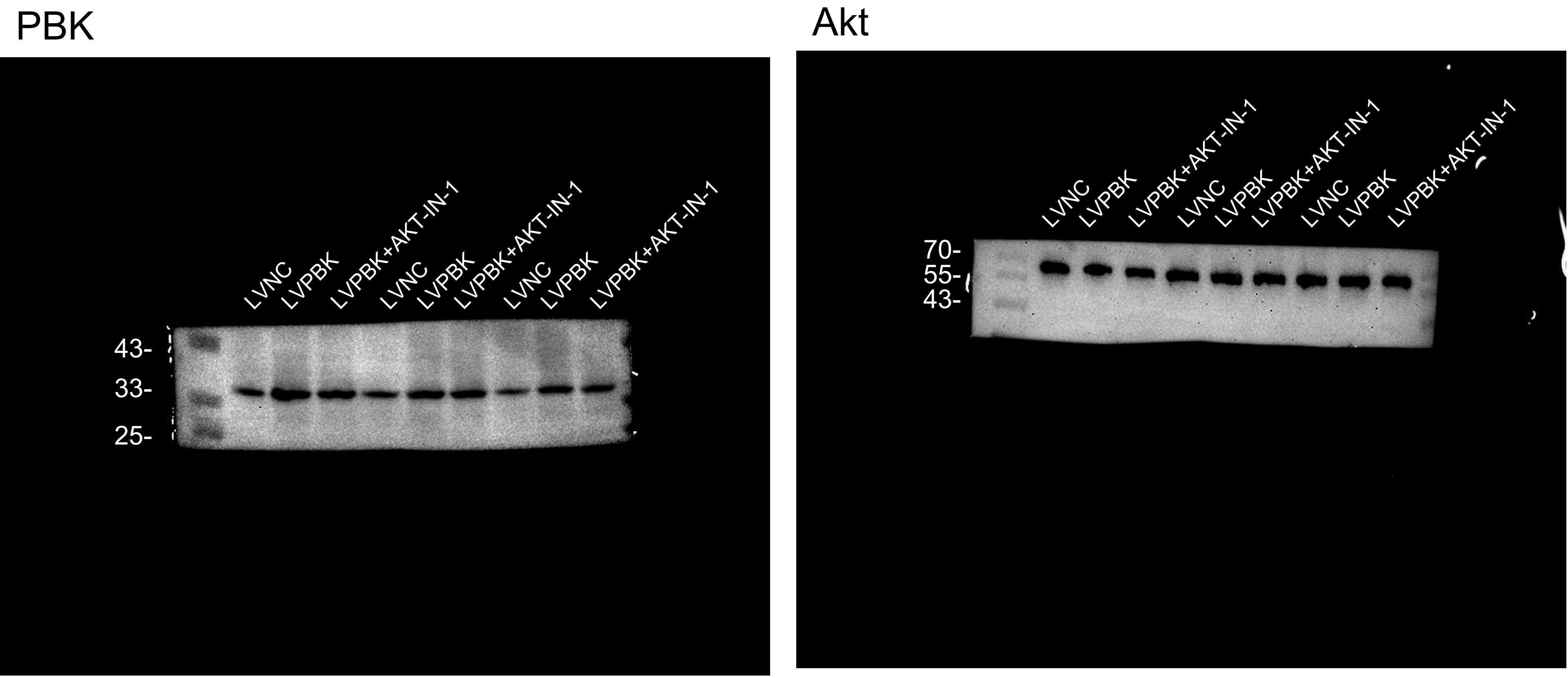
PBK
LVNC
LVPBK
LVPBK+AKT-IN-1
LVNC
LVPBK
LVPBK+AKT-IN-1
LVNC
LVPBK
LVPBK+AKT-IN-1
43-
33-
25-
Akt
LVNC
LVPBK
LVPBK+AKT-IN-1
LVNC
LVPBK
LVPBK+AKT-IN-1
LVNC
LVPBK
LVPBK+AKT-IN-1
70-
55-
43-

p-Akt

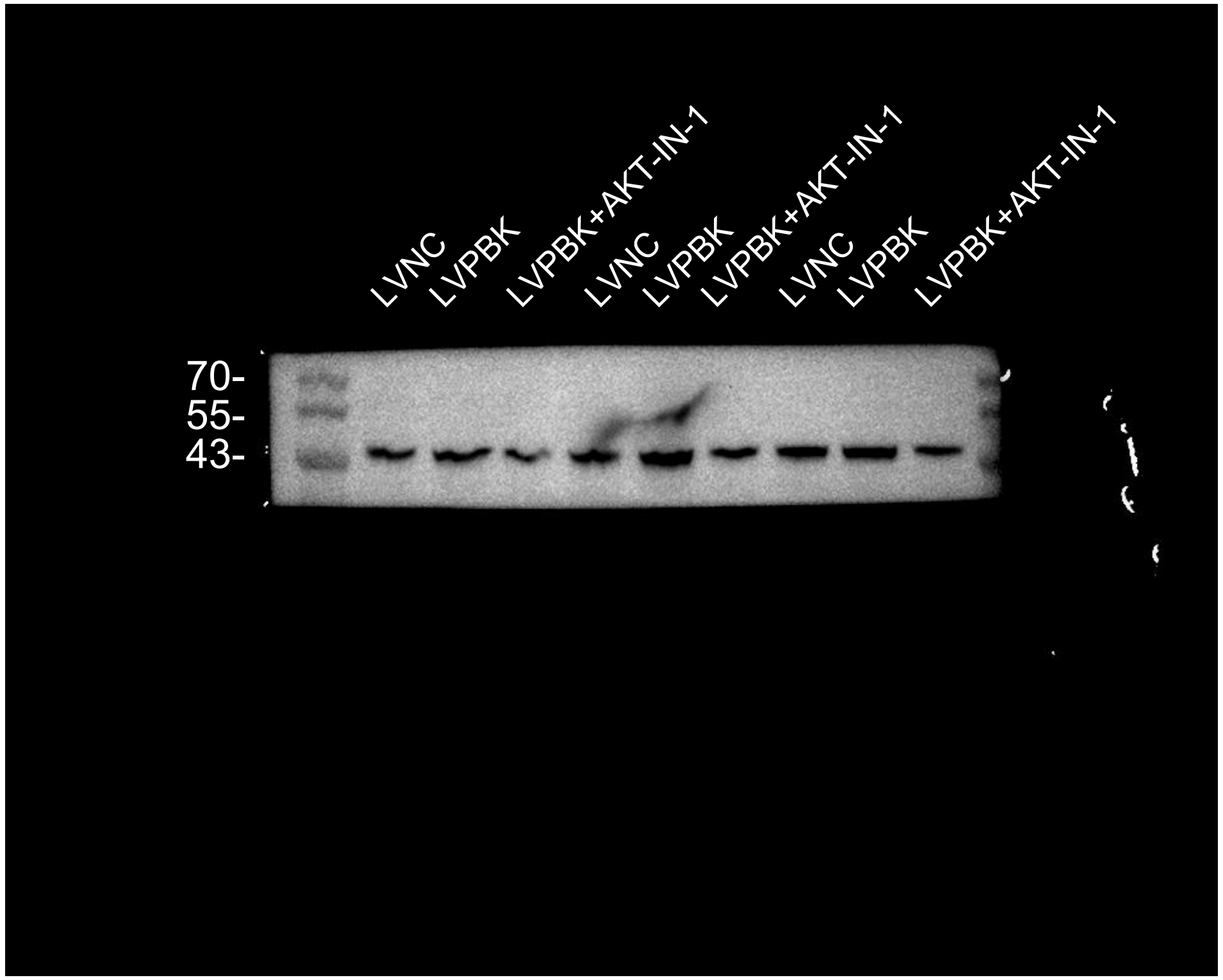
LVNC
LVPBK
LVPBK+AKT-IN-1
LVNC
LVPBK
LVPBK+AKT-IN-1
LVNC
LVPBK
LVPBK+AKT-IN-1
70-
55-
43-

GAPDH

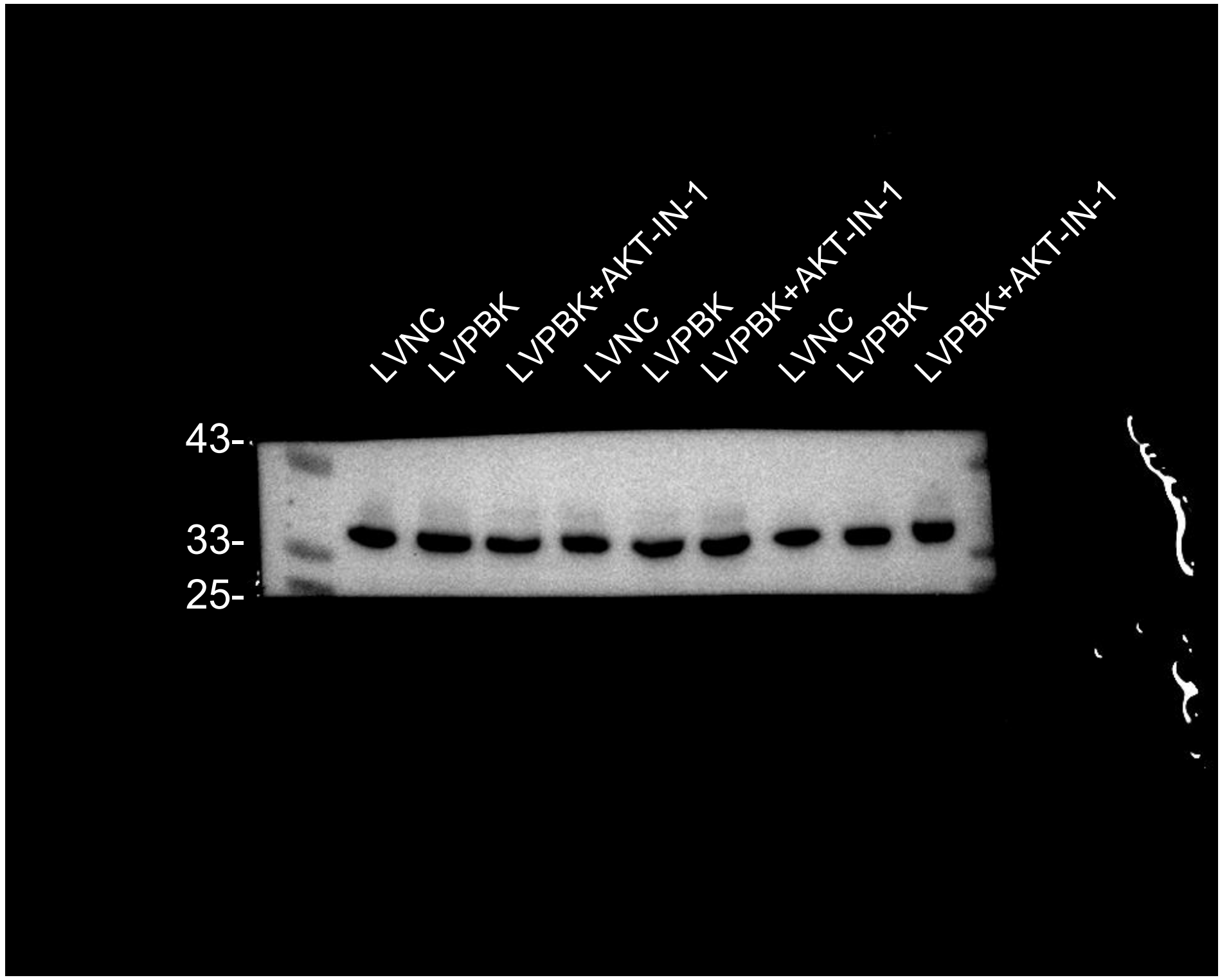
LVNC
LVPBK
LVPBK+AKT-IN-1
LVNC
LVPBK
LVPBK+AKT-IN-1
LVNC
LVPBK
LVPBK+AKT-IN-1
43-
33-
25-

Figure 8D

PBK

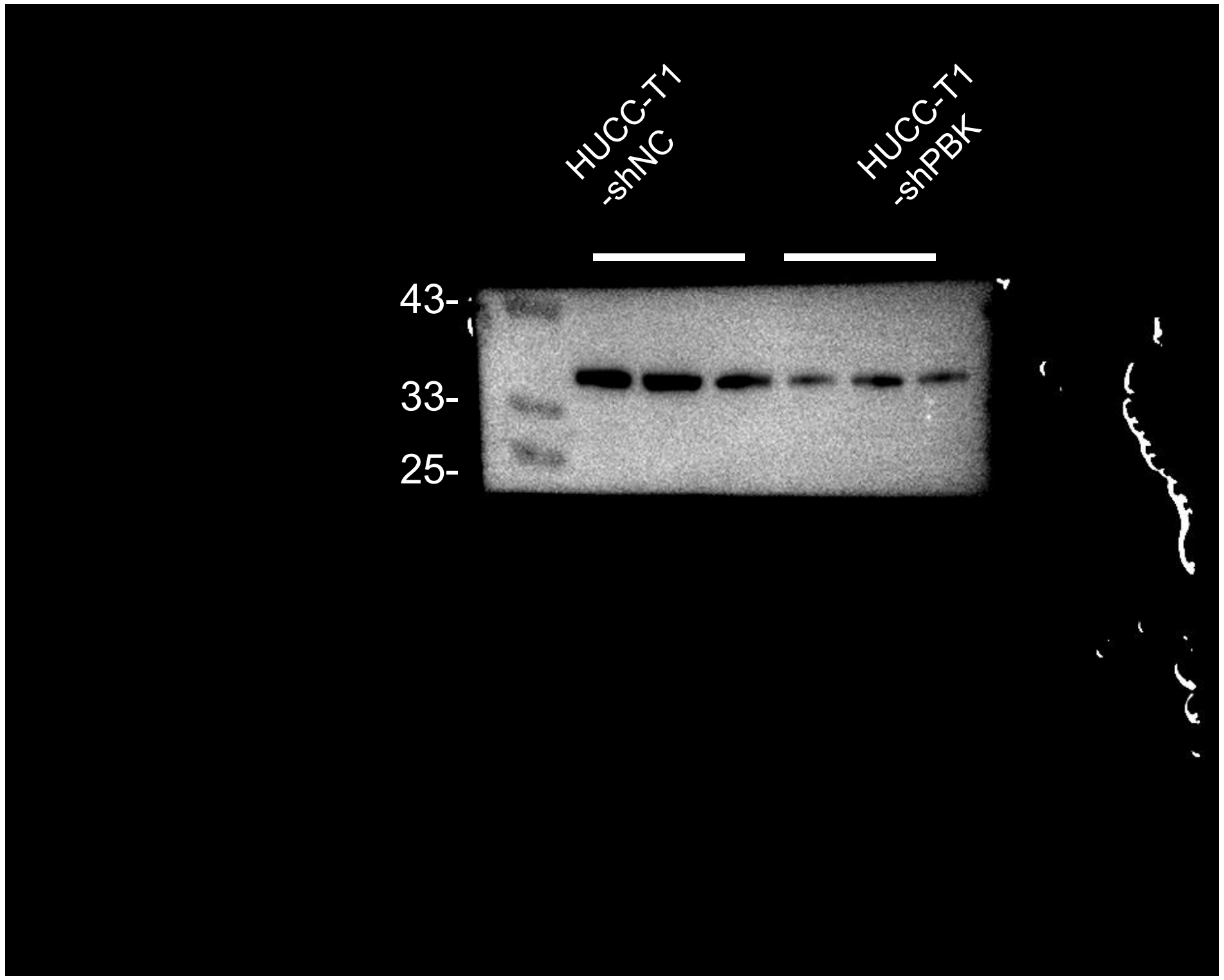

p-Akt

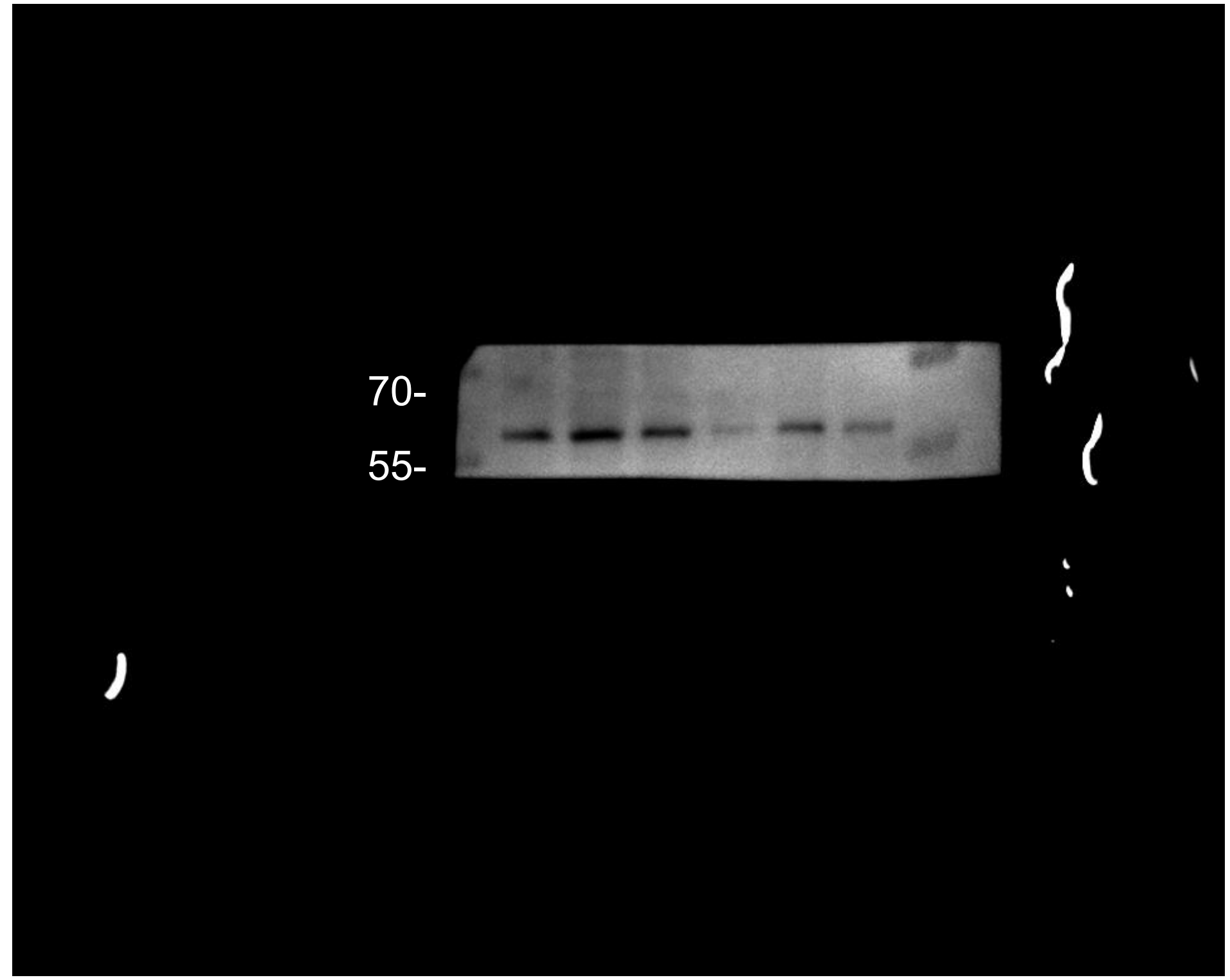

Akt

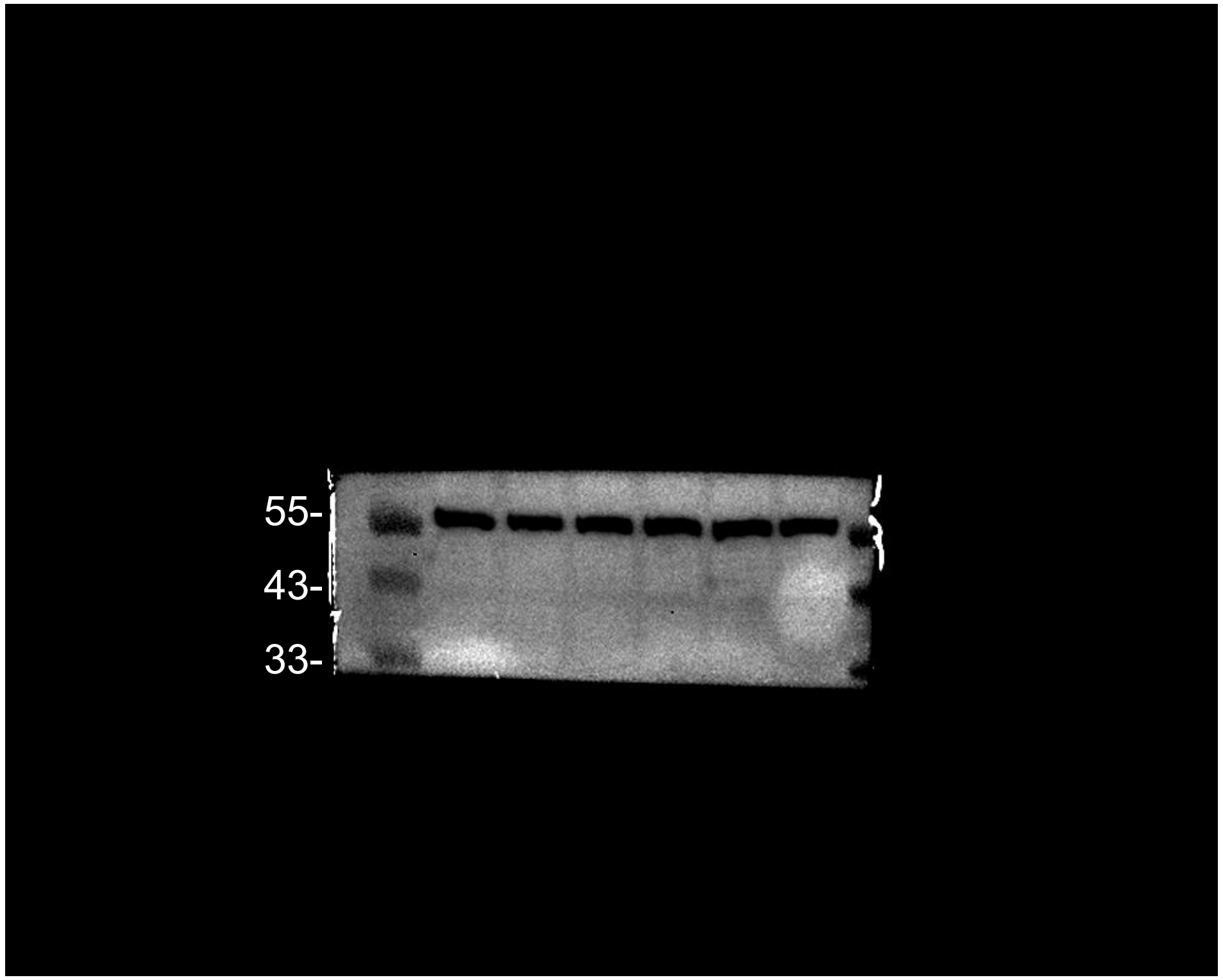

KI67

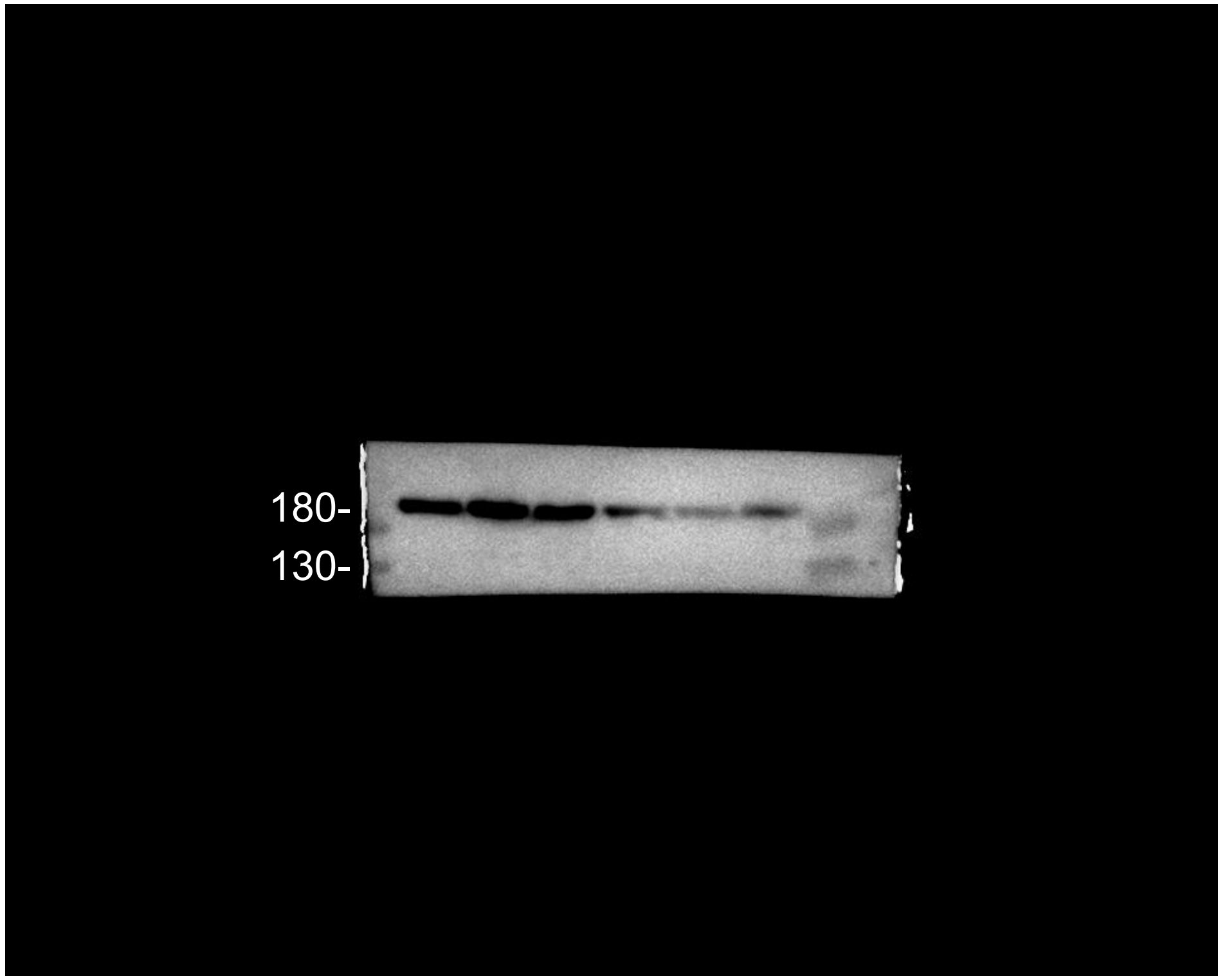

PCNA

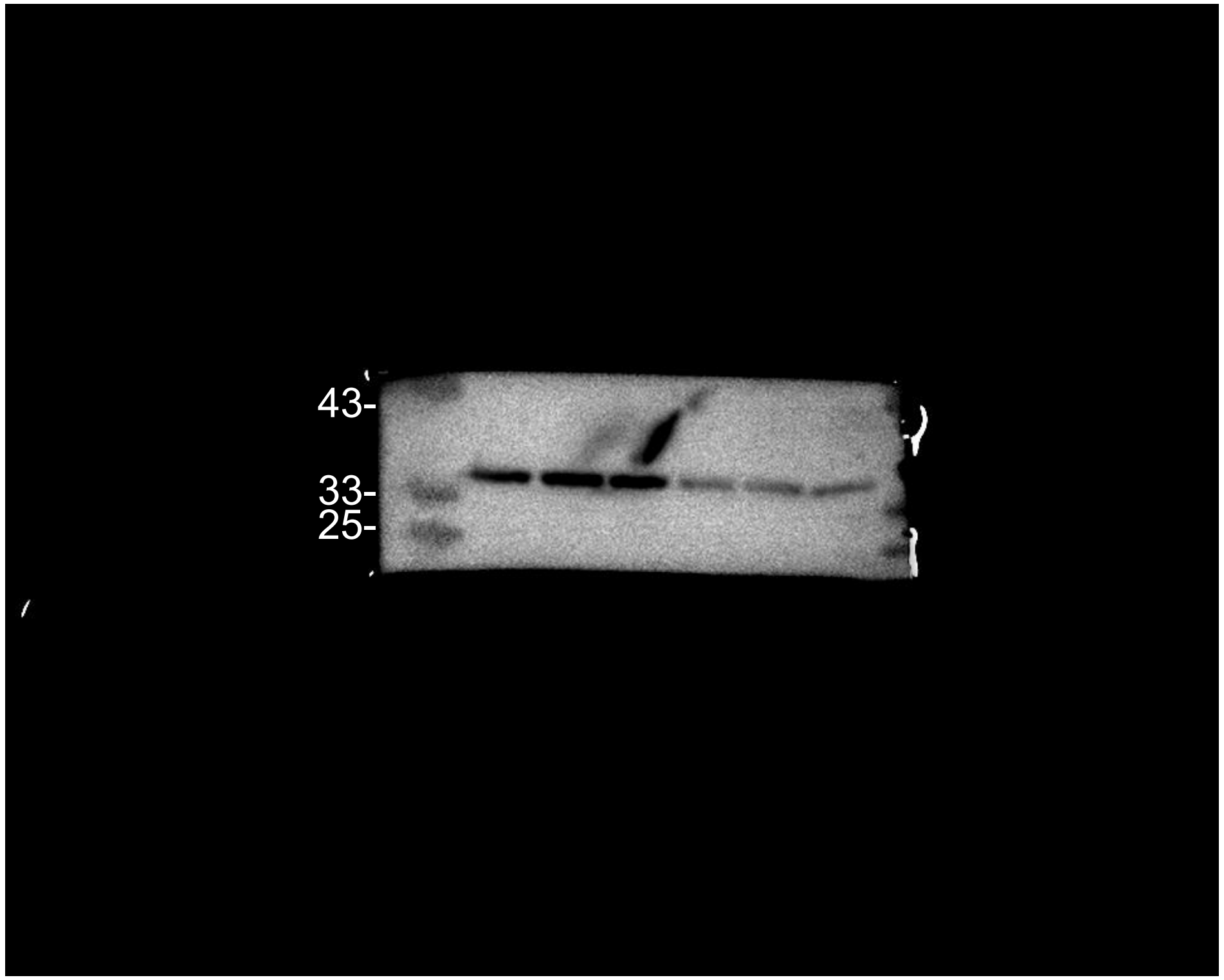

GAPDH

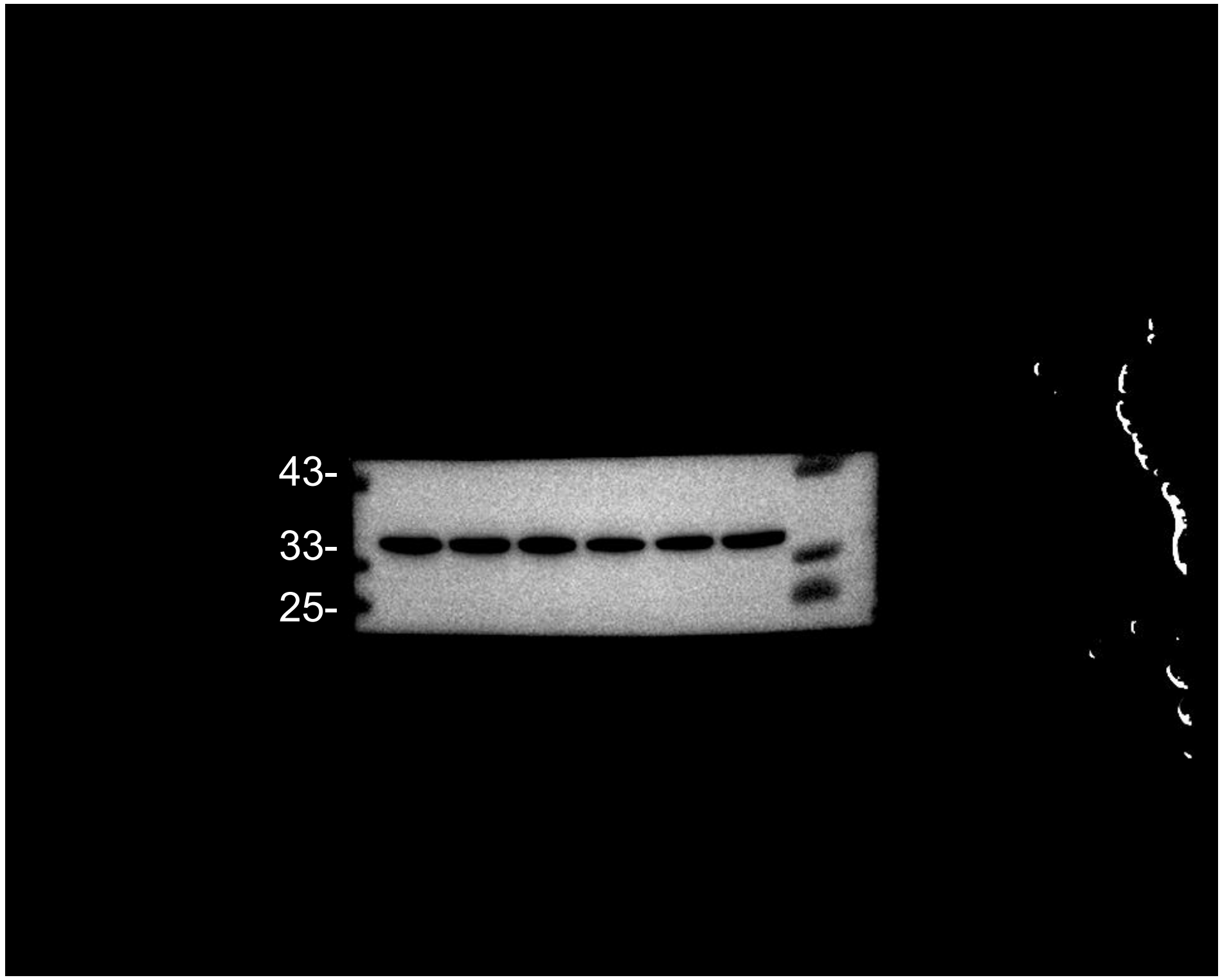

Supplement: Supplementary file 1 [file mmc5.pdf]
